# The ferroportin Q248H mutation protects from anemia, but not malaria or bacteremia

**DOI:** 10.1126/sciadv.aaw0109

**Published:** 2019-09-04

**Authors:** John Muthii Muriuki, Alexander J. Mentzer, Gavin Band, James J. Gilchrist, Tommy Carstensen, Swaib A. Lule, Morgan M. Goheen, Fatou Joof, Wandia Kimita, Reagan Mogire, Clare L. Cutland, Amidou Diarra, Anna Rautanen, Cristina Pomilla, Deepti Gurdasani, Kirk Rockett, Neema Mturi, Francis M. Ndungu, J. Anthony G. Scott, Sodiomon B. Sirima, Alireza Morovat, Andrew M. Prentice, Shabir A. Madhi, Emily L. Webb, Alison M. Elliott, Philip Bejon, Manjinder S. Sandhu, Adrian V. S. Hill, Dominic P. Kwiatkowski, Thomas N. Williams, Carla Cerami, Sarah H. Atkinson

**Affiliations:** 1 Kenya Medical Research Institute (KEMRI) Wellcome Trust Research Programme, Kilifi, Kenya.; 2 Wellcome Centre for Human Genetics, Nuffield Department of Medicine, University of Oxford, Oxford, UK.; 3 Department of Paediatrics, University of Oxford, Oxford, UK.; 4 Wellcome Sanger Institute, Hinxton, Cambridge, UK.; 5 Medical Research Council/Uganda Virus Research Institute and London School of Hygiene & Tropical Medicine Uganda Research Unit, Entebbe, Uganda.; 6 London School of Hygiene and Tropical Medicine, London, UK.; 7 Medical Research Council Unit The Gambia at London School of Hygiene and Tropical Medicine, Banjul, The Gambia.; 8 University of North Carolina School of Medicine, CB 7435, Chapel Hill, North Carolina USA.; 9 Medical Research Council: Respiratory and Meningeal Pathogens Research Unit, Faculty of Health Sciences, University of the Witwatersrand, Johannesburg, South Africa.; 10 Centre de Recherche Action en Sante (GRAS), 06 BP 10248, Ouagadougou 06, Burkina Faso.; 11 Department of Clinical Biochemistry, Oxford University Hospitals, Oxford, UK.; 12 Centre for Tropical Medicine and Global Health, Nuffield Department of Medicine, University of Oxford, Oxford, UK.; 13 Centre for Clinical Vaccinology and Tropical Medicine and the Jenner Institute Laboratories, University of Oxford, Oxford, UK.; 14 Department of Medicine, Imperial College, London, UK.

## Abstract

Iron acquisition is critical for life. Ferroportin (FPN) exports iron from mature erythrocytes, and deletion of the *Fpn* gene results in hemolytic anemia and increased fatality in malaria-infected mice. The *FPN* Q248H mutation (glutamine to histidine at position 248) renders FPN partially resistant to hepcidin-induced degradation and was associated with protection from malaria in human studies of limited size. Using data from cohorts including over 18,000 African children, we show that the Q248H mutation is associated with modest protection against anemia, hemolysis, and iron deficiency, but we found little evidence of protection against severe malaria or bacteremia. We additionally observed no excess *Plasmodium* growth in Q248H erythrocytes ex vivo, nor evidence of selection driven by malaria exposure, suggesting that the Q248H mutation does not protect from malaria and is unlikely to deprive malaria parasites of iron essential for their growth.

## Teaser

The FPN Q248H mutation protects children from anemia, hemolysis, and iron deficiency, but not malaria or bacterial infection.

## INTRODUCTION

Control of iron metabolism is fundamental to almost all known life. Malaria parasites and other infectious pathogens require iron to grow and multiply, and the human host has evolved to withhold iron from pathogens using iron-binding and chaperone transport proteins (*1*). Ferroportin (FPN), the sole known cellular iron exporter, is highly expressed in duodenal enterocytes, hepatocytes, and splenic macrophages to allow iron absorption, storage, and recycling (*2*). FPN is regulated by the iron hormone hepcidin in response to iron overload, erythropoiesis, and inflammation. Hepcidin reduces cellular iron export by inducing the internalization and degradation of FPN. A recent study showed that FPN is also abundantly expressed on mature red blood cells (RBCs) and that conditional deletion of the *Fpn* gene resulted in the accumulation of excess intracellular iron, hemolytic anemia, and increased parasitemia and death in malaria-infected mice (*3*).

**Fig. 1:**
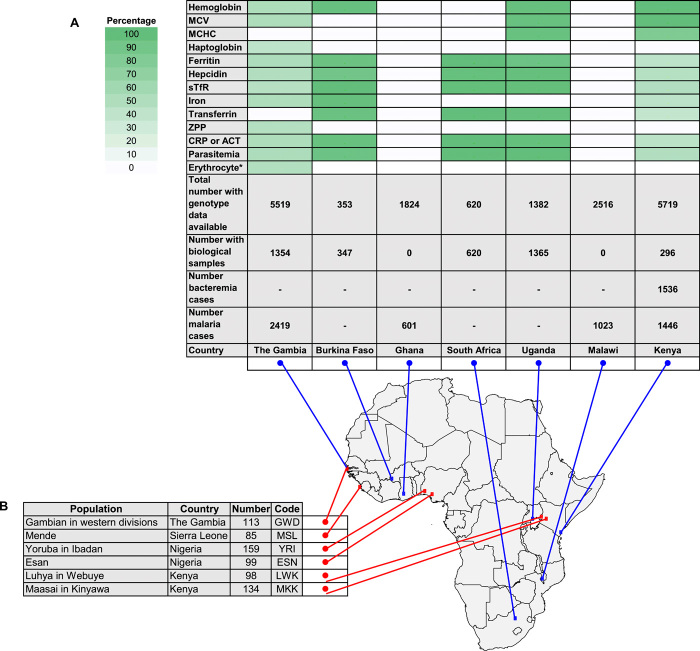
Collections with biological samples and phenotype and genetic data available in humans to test associations between anemia, iron, malaria, and bacteremia traits and the *FPN* Q248H mutation. (**A**) Proportion of total individuals with samples and data available for iron-related traits, and the Q248H mutation are shown in a heatmap of green gradient, representing the proportion of individuals with biological samples available, stratified by population with longitude and latitude represented on a map of the African continent. (**B**) Individuals with genetic data available from the 1000 Genomes Phase 3 Project with sequence data available stratified by population with longitude and latitude represented on a map of the African continent. MCV, mean cell volume; sTFR, soluble transferrin receptor; ZPP, zinc protoporphyrin; CRP, C-reactive protein; erythrocyte* includes samples available for ex vivo *Plasmodium* growth assay.

In humans, a mutation in *FPN*, Q248H (glutamine to histidine at position 248), increases cellular iron export by rendering FPN partially resistant to degradation by hepcidin, resulting in a 60% decrease in intracellular iron in Q248H cells (*4*). The effects of enhanced iron export may vary depending on cell type, for example, in erythrocytes, it might protect from excess toxic iron and hemolytic anemia (*3*), or in hematopoietic progenitor cells may reduce iron availability for erythropoiesis, and in enterocytes and macrophages, it might increase iron absorption and recycling. Alternately, Q248H has also been shown to impair cellular iron export [retaining only 25% of wild-type (WT) iron efflux] (*5*) or have no functional effect (*6*, *7*). Previous studies, with limited sample sizes, have found conflicting associations between Q248H and anemia or iron overload (table S1 and fig. S1). Moreover, by increasing cellular iron export, Q248H FPN could deprive intracellular pathogens, such as *falciparum* malaria or *Salmonella*, of iron, a nutrient critical for their growth. Since the Q248H mutation occurs only in African populations, it has been hypothesized that the variant has been positively selected due to protection from malaria (*3*). Using data from 18,320 children from across Africa and 380 pregnant women from The Gambia (Fig. 1), we tested the hypothesis that the Q248H mutation is associated with protection from hemolytic anemia, iron deficiency, and intracellular pathogens such as malaria or invasive *Salmonella*.

## RESULTS

### Characteristics of study participants

Figure 1 shows the populations included in this study. We tested associations with anemia and measures of iron status among 3374 children aged from 3 months to 7 years from community-based cohorts in Uganda, The Gambia, Burkina Faso, Kenya, and South Africa. Since sickness can alter measures of iron status, we selected healthy children living in the community with available stored samples. The Q248H mutation was observed among 10.5% of children, confirming its presence in African populations (*n* = 355 Q248H carriers, 342 heterozygotes and 13 homozygotes combined assuming a dominant mode of action). The characteristics of the community-based cohorts are summarized in table S2. For the ex vivo studies, RBCs came from anemic but otherwise healthy children (*n* = 229) and pregnant women (*n* = 380) from The Gambia. For the clinical studies, we analyzed case-control data from genome-wide association studies of severe malaria in 11,982 children, 5489 hospitalized patients with severe malaria (*n* = 658 Q248H carriers), and 6493 matched controls (*n* = 1519 Q248H carriers) from The Gambia, Malawi, Kenya, and Ghana (*8*). For bacteremia, we analyzed data from 1536 hospitalized Kenyan children with positive blood cultures (*n* = 223 Q248H) and 2677 matched community controls (*n* = 384 Q248H; Fig. 1 and Table 2) (*9*).

### *FPN* Q248H is associated with modest protection against anemia and iron deficiency

**Fig. 2:**
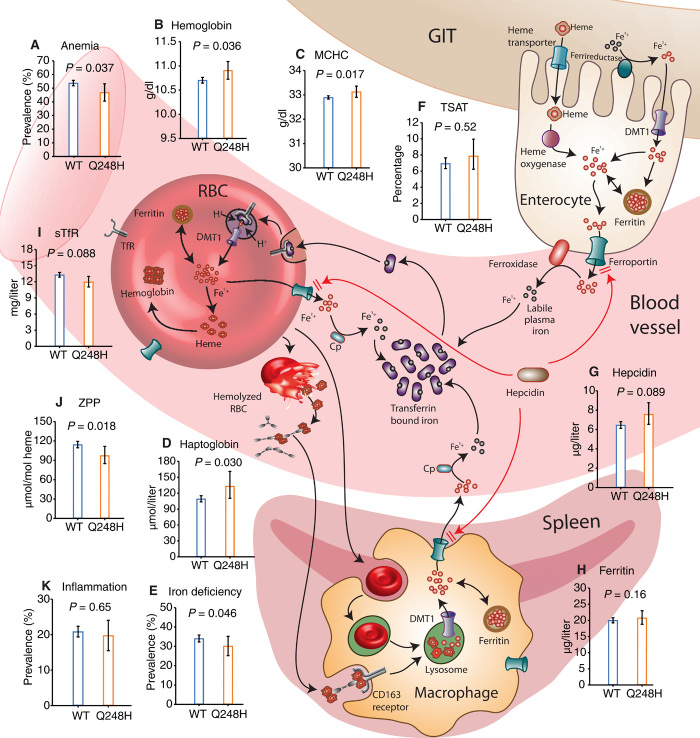
*FPN* Q248H mutation is associated with protection from anemia, red cell hemolysis, and iron deficiency. The Q248H mutation renders FPN partially resistant to hepcidin degradation (*4*), increasing iron export through enterocytes, erythrocytes, and macrophages. The effects of Q248H on (**A**) anemia, (**B**) hemoglobin, (**C**) MCHC, (**D**) haptoglobin, (**E**) iron deficiency, (**F**) transferrin saturation (TSAT), (**G**) hepcidin, (**H**) ferritin, (**I**) soluble transferrin receptors (sTfR), (**J**) ZPP, and (**K**) inflammation indicate reduced hemolysis, limited effect on iron status, and no association with inflammation. Plotted values are geometric means except anemia, iron deficiency, and inflammation, which are percentages. All *P* values were two tailed and derived from regression models adjusted for age, sex, and cohort. GIT, gastrointestinal tract; DMT1, divalent metal transporter 1.

We first evaluated the effect of the mutation on the risk of anemia in 2666 children with hemoglobin measurements. The prevalence of anemia (hemoglobin <11 g/dl) was lower in Q248H children (46.8%) compared with WT [53.7%; odds ratio (OR), 0.75; 95% confidence interval (CI), 0.57–0.98; *P* = 0.037; comparing Q248H heterozygote and homozygote carriers to noncarriers and using two-tailed tests throughout; Fig. 2A and Table 1]. Mean hemoglobin concentrations were 0.22 g/dl higher in Q248H children compared with WT (10.81 g/dl versus 10.59 g/dl; *P* = 0.036) (Fig. 2B and Table 1). The association between Q248H and hemoglobin concentrations was consistent across cohorts and by gender (figs. S2 and S3). A meta-analysis of the current and previous studies and of the individual cohorts showed that the mutation is associated with a significant increase in hemoglobin concentration (figs. S1 and S2). We similarly observed higher mean corpuscular hemoglobin concentrations (MCHCs) in children carrying Q248H compared with WT (*P* = 0.017, Fig. 2C), but no evidence of a difference in mean corpuscular volume (Table 1). The low frequency of Q248H homozygotes makes it difficult to distinguish heterozygote and homozygote effects in these data, but estimated effects appear broadly consistent with the dominance model assumed above, with some exceptions that will require additional data to resolve (table S3). We hypothesized that the modestly increased hemoglobin concentrations and MCHCs observed in Q248H carriers could be due to reduced hemolysis and/or improved iron status.

**Table 1:** The *FPN* Q248H mutation is associated with protection from anemia, hemolysis, and iron deficiency in community-based children. Analyses were adjusted for age, sex, and country. CRP, C-reactive protein; MCV, mean corpuscular volume; sTfR, soluble transferrin receptor; TSAT, transferrin saturation; ZPP, zinc protoporphyrin.

		Q248H*		**WT**			
**Category**	**All, *n***	**Q248H, *n***	***n* (%)**	**WT, *n***	***n* (%)**	**OR (95% CI)**	***P***
Anemia^†^	2666	237	111 (46.8)	2429	1305 (53.7)	0.75 (0.57 to 0.98)	0.037
Iron deficiency^‡^	3065	325	98 (30.2)	2740	933 (34.1)	0.77 (0.60 to 0.99)	0.046
Iron deficiency anemia^§^	2363	208	35 (16.8)	2155	435 (20.2)	0.77 (0.52 to 1.14)	0.19
Inflammation^║^	3149	334	66 (19.8)	2815	587 (20.9)	0.94 (0.70 to 1.25)	0.65
**Biomarkers**	**All, *n***	**Q248H, *n***	**Geometric mean (SD)**	**WT, *n***	**Geometric mean (SD)**	**Estimate** **(95% CI)**	***P***
Hemoglobin, g/dl	2666	237	10.81 (1.15)	2429	10.59 (1.16)	0.21 (0.01 to 0.40)	0.036
MCHC, g/dl	1537	131	33.10 (1.04)	1406	32.87 (1.04)	0.27 (0.05 to 0.50)	0.017
MCV, fL	2324	205	72.98 (1.12)	2119	72.76 (1.12)	0.16 (−0.87 to 1.19)	0.76
Haptoglobin, μmol/liter	587	50	133.22 (1.98)	537	109.32 (1.87)	0.20 (0.02 to 0.38)	0.030
Ferritin, μg/liter	3065	325	20.80 (2.53)	2740	20.03 (2.84)	0.08 (−0.03 to 0.20)	0.16
Hepcidin, μg/liter	3080	319	8.31 (3.48)	2761	7.33 (3.87)	0.14 (−0.02 to 0.30)	0.089
sTfR, mg/liter	3091	325	11.98 (1.66)	2766	13.30 (1.67)	−0.07 (−0.16 to 0.01)	0.088
Iron, μmol/liter	459	45	5.45 (1.91)	414	4.83 (2.35)	0.14 (−0.10 to 0.37)	0.25
Transferrin, g/liter	2380	258	2.62 (1.28)	2122	2.70 (1.27)	−0.06 (−0.14 to 0.02)	0.14
TSAT, %	430	40	7.89 (2.13)	390	6.95 (2.62)	0.09 (−0.18 to 0.36)	0.52
ZPP, μmol/mol heme	684	66	97.43 (1.80)	618	114.63 (1.77)	−0.17 (−0.31 to −0.03)	0.018
CRP, mg/liter	2403	263	1.08 (5.07)	2140	1.35 (5.44)	−0.19 (−0.41 to 0.02)	0.079
ACT, g/liter	737	68	0.47 (1.34)	669	0.44 (1.30)	0.05 (−0.01 to 0.12)	0.12

*Heterozygotes and homozygotes.

†Anemia was defined as hemoglobin <11 g/dl.

‡Iron deficiency was defined as plasma ferritin <12 or <30 μg/liter in the presence of inflammation in children <5 years or <15 μg/liter in children ≥5 years.

§Iron deficiency anemia was defined as iron deficiency with anemia.

║Inflammation was defined as C-reactive protein >5 mg/liter or ACT >0.6 g/liter in The Gambia.

To test the hypothesis that higher hemoglobin concentrations among Q248H children may be due to reduced hemolysis, we measured haptoglobin concentrations in 587 Gambian children (2 to 6 years). We observed that Q248H carriers (*n* = 50) had higher geometric mean haptoglobin concentrations compared with WT (133.22 μmol/liter versus 109.32 μmol/liter; β = 0.20; 0.02, 0.38; *P* = 0.03), consistent with reduced hemolysis in Q248H children (Fig. 2D and Table 1). These findings support the hypothesis that reduced hemolysis may contribute to the higher hemoglobin concentrations observed in Q248H children.

We further hypothesized that higher hemoglobin levels could be due to enhanced absorption and recycling of iron among Q248H carriers, leading to improved iron status as previously observed in smaller studies (table S1). Among 3065 children, 1031 (33.6%) were iron deficient. The Q248H variant (*n* = 325) was associated with a 23% reduction in the odds of iron deficiency (*P =* 0.046; comparing Q248H heterozygotes and homozygotes to noncarriers) and iron deficiency anemia (*P =* 0.19; Table 1). We observed a trend toward improved iron status for individual measures of iron status among Q248H children; however, with the exception of zinc protoporphyrin, a sensitive indicator of iron deficiency (*P* = 0.018), none of the associations reached conventional statistical significance (Fig. 2 and Table 1). Increased hepcidin expression is a major factor in the alteration of iron stores during inflammation [defined as C-reactive protein >5 mg/liter or α_1_-antichymotrypsin (ACT) >0.6 g/liter], and Q248H FPN may be partially resistant to hepcidin (*4*); thus, in secondary analyses, we stratified by inflammation. The prevalence of inflammation did not differ between Q248H and WT children (*P* = 0.65; Fig. 2K and Table 1). After excluding children with inflammation, we observed a 30% reduction in the odds of iron deficiency among Q248H carriers [*n* = 2409; adjusted OR, 0.7; 0.5–0.9; *P* = 0.018; fig. S3C] and a significant increase in ferritin concentrations (β = 0.1; 0.01–0.2; *P* = 0.037). We found no significant interaction with inflammation or gender in predicting iron status (fig. S3). We further stratified by low and high hepcidin (using a threshold of 5.5 ng/ml) but found no evidence of an interaction. Together, our findings suggest, in agreement with previous studies (table S1), that the Q248H mutation is associated with modestly improved iron status in African children.

### Little evidence that *FPN* Q248H is associated with protection from malaria or bacteremia

**Fig. 3:**
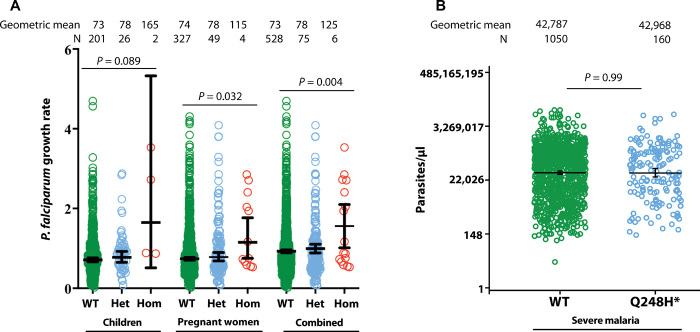
Effect of *FPN* Q248H variant on parasite *P. falciparum* growth rates and parasite density. (**A**) Ex vivo parasite growth rates in fresh erythrocytes from Gambian children (6 to 29 months) and mid-pregnant women stratified by carriage of Q248H *FPN* mutation (Het, heterozygous; Hom, homozygous). Parasite growth rates are geometric means from log-transformed data from triplicate assays. See the Materials and Methods for details of the assays and the clinical trials from which the samples were derived. A total of 229 genotyped children contributed data on up to three visits each (days 0, 49, and 84 of iron supplementation) and 380 genotyped pregnant women contributed data on up to four visits each (days 0, 14, 49, and 84). *P* values were calculated by analysis of variance (ANOVA) with visit day as a covariate to allow for supplementation effect. There was no interaction with hemoglobin or anemia or with plasma hepcidin (as a continuous variable or as above or below the thresholds of 5.5 ng/ml in children and 2.5 ng/ml in pregnant women). In the combined analysis of women and children, there was no interaction with study, confirming that merging of the datasets was legitimate. (**B**) *P. falciparum* parasite densities in hospitalized Kenyan children with severe malaria stratified by carriage of Q248H. Parasite densities are geometric means from log-transformed data. N, severe malaria cases. *P* values were derived from regression models adjusted for age, sex, and cohort. *Q248H heterozygotes and homozygotes combined (*n* = 3 homozygotes).

It has been proposed that the *FPN* Q248H variant protects individuals from malaria (*3*). We used cell culture methods to evaluate *Plasmodium falciparum* growth rates in fresh washed erythrocytes from 229 Gambian children (6 to 27 months, 28 with the Q248H variant, of whom 2 were homozygous) and 380 mid-pregnant women (53 with Q248H, of whom 4 were homozygous) participating in randomized controlled trials of hepcidin-guided iron supplementation (*10*, *11*). Erythrocytes from carriers of the Q248H variant consistently supported slightly faster parasite growth than WT (*P* = 0.089 in children, *P* = 0.032 in pregnant women, and *P* = 0.004 for the women and children combined) (Fig. 3A). To test the hypothesis that Q248H would offer protection in the face of raised hepcidin (*3*), we subdivided by high and low plasma hepcidin. There was no significant interaction.

We then evaluated whether the Q248H variant protects children from malaria in clinical studies (*8*). The Q248H mutation was not significantly associated with severe malaria overall (e.g., OR, 0.91; 0.81–1.01; for an effect of one or two copies of Q248H relative to WT; *P* = 0.08) or in individual cohorts (Table 2). Moreover, in subgroup analyses, the Q248H mutation was not significantly associated with cerebral malaria (OR, 0.90; 0.77–1.06; estimated across cohorts), severe malarial anemia (OR, 0.90; 0.71–1.16), or mortality (OR, 1.00; 0.78–1.29; Table 2). Inclusion of genotypes at the sickle cell causing variant rs334, which is strongly associated with malaria susceptibility in these populations, did not affect these results (table S4). Moreover, parasite density did not differ by the Q248H mutation (Fig. 3B). The prevalence of mild malaria among community-based children also did not differ by Q248H (Table 2).

**Table 2:** Little evidence that *FPN* Q248H provides protection against severe malaria or invasive bacterial infection. All *P* values reflect two-tailed tests. NTS, nontyphoidal *Salmonella*; RR, relative risk.

**Outcome**	**All**	Q248H*	**WT**	**RR/OR** **(95% CI)**	***P***
Malaria susceptibility^†^					
Mild malaria	260/2,550 (10.2%)	25/232 (10.8%)	235/2,318 (10.1%)	1.06 (0.68–1.64)	0.79
Severe malaria^‡^	5,489/11,982 (45.8%)	658/1519 (43.3%)	4,831/10,463(46.1%)	0.91 (0.81–1.01)	0.08
(The Gambia^§^)	2,419/4,910 (49.3%)	225/479 (47.0%)	2,194/4,431 (49.5%)	0.88 (0.73–1.07)	0.21
(Malawi^§^)	1,023/2,345 (43.6%)	174/419 (41.5%)	849/1,926 (44.1%)	0.89 (0.72–1.10)	0.28
(Kenya^§^)	1,446/2,924 (49.5%)	191/405 (47.2%)	1,255/2,519 (49.8%)	0.98 (0.79–1.22)	0.87
(Ghana^║^)	601/1,803 (33.3%)	68/216 (31.5%)	533/1,587 (33.6%)	0.83 (0.59–1.17)	0.29
					
Cerebral malaria^‡^	1,948/7,971 (24.4%)	249/1043 (23.9%)	1,699/6,928 (24.5%)	0.90 (0.77–1.06)	0.21
(The Gambia^§^)	758/3,676 (20.6%)	60/356 (16.9%)	698/3,320 (21.0%)	0.73 (0.55–0.99)	0.04
(Malawi^§^)	644/2,057 (31.3%)	116/373 (31.1%)	528/1,684 (31.4%)	0.95 (0.75–1.22)	0.71
(Kenya^§^)	546/2,238 (24.4%)	73/314 (23.2%)	473/1,924 (24.6%)	1.00 (0.75–1.34)	0.99
					
Severe malarial anemia^‡^	735/7,971 (9.2%)	82/1043 (7.9%)	653/6,928 (9.4%)	0.90 (0.71–1.16)	0.42
(The Gambia^§^)	428/3,676 (11.6%)	43/356 (12.1%)	385/3,320 (11.6%)	0.96 (0.68–1.35)	0.82
(Malawi^§^)	91/2,057 (4.4%)	12/373 (3.2%)	79/1,684 (4.7%)	0.69 (0.37–1.29)	0.24
(Kenya^§^)	216/2,238 (9.7%)	27/314 (8.6%)	189/1,924 (9.8%)	0.93 (0.60–1.43)	0.74
					
Malaria-related death^‡^	677/4,669 (14.5%)	86/571 (15.1%)	591/4,098 (14.4%)	1.00 (0.78–1.29)	0.99
(The Gambia^§^)	309/2,335 (13.2%)	27/219 (12.3%)	282/2,116 (13.3%)	0.91 (0.59–1.39)	0.65
(Malawi^§^)	200/1,018 (19.6%)	37/173 (21.4%)	163/845 (19.3%)	1.14 (0.76–1.71)	0.51
(Kenya^§^)	168/1,316 (12.8%)	22/179 (12.3%)	146/1,137 (12.8%)	0.94 (0.58–1.52)	0.80
					
Bacteremia^¶^	1,536/4,213 (36.5%)	223/607 (36.7%)	1,313/3,606 (36.4%)	1.04 (0.87–1.24)	0.68
*Streptococcus pneumoniae*	426/4,213 (10.1%)	57/607 (9.4%)	369/3,606 (10.2%)	0.93 (0.69–1.26)	0.65
NTS	180/4,213 (4.3%)	31/607 (5.1%)	149/3,606 (4.1%)	1.25 (0.83–1.87)	0.28
*Escherichia coli*	151/4,213 (3.6%)	26/607 (4.3%)	125/3,606 (3.5%)	1.27 (0.82–1.97)	0.29
*Haemophilus influenzae*	128/4,213 (3.0%)	12/607 (2.0%)	116/3,606 (3.2%)	0.64 (0.35–1.18)	0.15
*Staphylococcus aureus*	175/4,213 (4.2%)	20/607 (3.3%)	155/3,606 (4.3%)	0.80 (0.50–1.30)	0.37

*Heterozygotes and homozygotes.

†Mild malaria was defined as *P. falciparum* positive slide measured in community-based cohorts in Uganda, The Gambia, Burkina Faso, and Kenya. Severe malaria was defined as positive for *P. falciparum* parasites and clinical features of severe malaria (*8*), including diagnosis of cerebral malaria, severe malarial anemia, and other clinical symptoms.

‡Relative risk, CI, and *P* value were computed by fixed-effect meta-analysis of estimates from the three case-control cohorts and (where applicable) the Ghanaian trios.

§Association analysis estimated by logistic regression adjusted for the first five principal components. For severe malaria and malaria-related death, results reflect binomial logistic regression of the phenotype compared with controls. For severe malaria subphenotypes, results reflect multinomial logistic regression of cerebral malaria, severe malarial anemia, and other severe malaria cases compared with controls. A dominant mode of effect is assumed.

║Counts reflect numbers of probands (affected children) and parents in 608 Ghanaian trios. Relative risk, CI, and *P* value are computed using a transmission disequilibrium test. A dominant mode of effect is assumed.

¶Bacteremia was defined as positive blood culture from hospitalized admission in Kilifi County Hospital in Kenya. Association analysis for all-cause bacteremia was estimated by logistic regression adjusted for sex and the first two principal components of genome-wide genotyping data to account for population structure. Pathogen-specific *P* values and odds ratios were derived by multinomial logistic regression adjusted for sex and population structure.

We further hypothesized that Q248H, by reducing intracellular iron, might protect from other intracellular pathogens such as nontyphoidal *Salmonella*, a common cause of mortality among African children (*12*). Among 1536 hospitalized Kenyan children with positive blood cultures and 2677 matched community controls (*9*), we found that the Q248H mutation was not associated with protection from all-cause bacteremia (OR, 1.05; 0.88–1.26) or with protection from bacteremia due to nontyphoidal *Salmonella* (OR, 1.25; 0.84–1.98), *Streptococcus pneumoniae*, *Escherichia coli*, *Haemophilus influenzae*, or *Staphylococcus aureus* (Table 2 and table S4).

### No evidence of *FPN* Q248H natural selection driven by malaria exposure

**Fig. 4:**
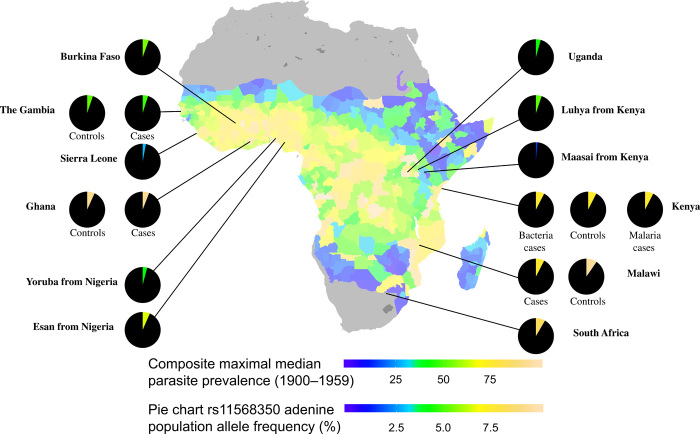
Frequencies of adenine allele of rs11568350 encoding the Q248H mutation in African populations compared with the composite maximal estimate of *P. falciparum* transmission in 2- to 10-year-olds between the years 1900 and 1959. Regional median estimates of transmission are provided across malaria endemic regions of the natural extent of sub-Saharan Africa including the Island of Madagascar as obtained from Snow *et al.* (*14*). Population-specific frequencies of the variant encoding the Q248H allele are shown as pie charts for each of the included populations with the more common ancestral allele colored in black and the rarer adenine allele colored. For illustration purposes, colors are assigned on a scale matched to the *P. falciparum* parasite prevalence scale using a frequency of 10% as equivalent to a prevalence of 1 (for example, green represents 5% allele frequency). If there were a correlation between the allele frequencies and malaria prevalence, one would expect to see similar colors in the pie chart slices and map contours. The allele frequencies and estimated maximal estimates of transmission per population are plotted against each other in fig. S4.

We used data from the 1000 Genomes Project (*13*) to confirm that the DNA variant (rs11568350) encoding for *FPN* Q248H was only observed in populations with African ancestry (Fig. 4). We found that the derived allele (adenine, encoding the histidine amino acid) varied from 1.5% (in the Kenyan Maasai population) to 9.6% (in Malawian individuals). We observed no evidence of deviation from the Hardy-Weinberg equilibrium (*P* = 0.079 to 1; table S5) or of significant levels of differentiation between populations [Fixation index (*F*_ST_) range, 0 to 0.06]. We further compared the frequencies of the derived allele in African populations against estimates of maximal rates of *P. falciparum* infection prevalence in children aged 2 to 10 years (Fig. 4) (*14*). We observed no correlation between the observed allele frequencies and parasite prevalence [Spearman rho = 0.27 between rs11568350 adenine allele and the maximum estimated prevalence between the years 1900 and 1959, *P* = 0.37 (fig. S4A); or between the years 2000 and 2015, rho range = −0.17 to 0.25, *P* = 0.34 to 0.97 (fig. S4B)], and one of the highest observed frequencies was in the South African population (8.4%), which has historically experienced low malaria transmission. Moreover, we noted the existence of a large number of other alleles across the genome in African populations with frequency characteristics equivalent to the FPN mutation (table S6).

## DISCUSSION

The *FPN* Q248H variant is prevalent in African populations and is thought to have been naturally selected to confer protection against malaria and other intracellular infections by increasing iron efflux from erythrocytes (*3*). Here, we report findings from large-scale genomic datasets evaluating the effect of the Q248H mutation on anemia, iron status, malaria, and bacterial infections. We found that the mutation was associated with modest protection against anemia and iron deficiency but found little evidence that the mutation was associated with protection against malaria or invasive bacterial infections. We further showed that the Q248H mutation does not appear to have been selected in African populations due to malaria exposure.

The Q248H mutation was associated with a modest reduction in the risk of anemia and modestly improved hemoglobin concentrations. The Q248H variant has previously been associated with trends toward both lower and higher hemoglobin concentrations in studies of small sample size (fig. S1 and table S1). A meta-analysis of the current and previous studies indicates an overall increase in hemoglobin concentrations in Q248H carriers (fig. S1). Higher hemoglobin concentrations might be due to higher iron efflux from Q248H erythrocytes protecting the cell from hemolysis (*3*). We observed higher concentrations of haptoglobin among Q248H carriers, indicating reduced levels of hemolysis. These findings are consistent with a previous report in a mouse model, which demonstrated that FPN, by reducing intracellular accumulation of iron and oxidant species, prevents hemolytic anemia and maintains red blood cell integrity (*3*). We further hypothesized that reduced hepcidin-induced degradation of FPN among Q248H carriers might lead to improved iron absorption by duodenal enterocytes and iron recycling by macrophages. Consistent with this hypothesis, we observed a trend toward improved iron status among children carrying the mutation corroborating findings from other studies (*15*–*18*). Zinc protoporphyrin levels were also significantly lower among Q248H individuals, suggesting no evidence for iron-deficient erythropoiesis, as previously suggested (*3*).

It has been proposed that *FPN* Q248H protects from malaria by depriving the parasite of iron within the red cell (*3*). However, we did not find a significant association between the mutation and malaria susceptibility in ex vivo or clinical studies. We found, instead, that erythrocytes from Q248H carriers supported slightly faster *P. falciparum* growth rates in ex vivo studies. Together, our data in large-scale studies suggest that any protection afforded by the Q248H mutation in natural populations is weak, with previous estimates of effect size (*3*) not generalizing to the phenotypes and populations considered here. In addition, the current study included 658 Q248H children with severe malaria, in contrast to a previous report with a limited sample size of 13 Q248H children with uncomplicated malaria and 11 Q248H women with placental malaria (*3*). These findings suggest that erythrocyte iron may not be an important source of iron for the malaria parasite.

Further, the *FPN* Q248H mutation was not associated with protection against all-cause bacteremia. Since the mutation may reduce intracellular iron, we specifically aimed to investigate whether the mutation is associated with susceptibility to nontyphoidal *Salmonella*, an intracellular pathogen, but found no evidence of protection, suggesting that nontyphoidal *Salmonella* may acquire iron through other mechanisms. Previous studies have found either no association or increased susceptibility to tuberculosis (another intracellular infection) in the Q248H mutation compared with the WT in small cohorts (*19*, *20*).

Given the lack of evidence for association between the Q248H mutation and multiple severe infectious diseases, we reevaluated the evidence that natural selection has driven the evolution of this variant (*3*). We found no correlation between the *FPN* Q248H derived allele and autochthonous *P. falciparum* prevalence. For instance, the derived allele frequency was highest in South Africa, a malaria-free population, thus suggesting that *P. falciparum* prevalence in the population is not associated with the mutation and arguing against selective pressure due to malaria. The low observed frequency of the Q248H allele outside Africa may have resulted from the loss of neutral allelic diversity that occurred as humans expanded out of Africa (*21*) rather than through a natural selection process. Together, these results provide little evidence for selection or for a relationship between the historical burden of malaria infection and the frequency of the Q248H mutation among populations.

In conclusion, using large-scale genomic datasets from African populations, we found that the Q248H mutation was associated with modest reductions in the risk of anemia and iron deficiency, which may be putatively due to reduced hemolysis and modestly improved iron status in Q248H carriers. These findings in large epidemiological studies build support for proposed biological pathways observed in mouse models (*3*), although the observed effect sizes are small and may not be clinically significant at an individual level. Last, we found little evidence that the Q248H mutation protects against malaria in ex vivo studies or against malaria or bacteremia in clinical studies and, finally, no evidence of positive selection of the variant attributable to malaria exposure.

## MATERIALS AND METHODS

### Study populations

The samples and data used in these analyses were sourced from multiple individual studies, many of which have data already available to general researchers. Participants from three countries (Uganda, South Africa, and Burkina Faso) were recruited into the VaccGene study with a primary aim of identifying genetic variants associated with differential response to vaccination in infancy but with ethical approval to undertake analyses investigating the effect of iron metabolism on susceptibility to infection. Individuals from each of the individual populations were included if their dates of birth, vaccination, and blood sampling were available and if it was confirmed that they had received three doses of routine vaccines and a single dose of measles vaccine by 12 months of age. Children were not further excluded on the basis of gender, ethnicity, HIV exposure, or any other health status. A range of clinical and demographic metadata were collected from the three cohorts, including the number of illnesses during the first year of life, details regarding the pregnancy and parental occupations, and self-reported ethnicities. A more detailed description of each of the populations follows below.

#### Uganda: The Entebbe Mother and Baby Study

The Entebbe Mother and Baby Study (EMaBS) is a prospective birth cohort that was originally designed as a randomized controlled trial to test whether anthelminthic treatment during pregnancy and early childhood was associated with differential response to vaccination or incidence of infections such as pneumonia, diarrhea, or malaria (http://emabs.lshtm.ac.uk/) (*22*). EMaBS originally recruited 2507 women; 2345 live births were documented, and 2115 children were still enrolled at 1 year of age. Pregnant women in the second or third trimester were enrolled at the Entebbe Hospital antenatal clinic if they were residents in the study area, planning to deliver in the hospital, willing to know their HIV status, and willing to take part in the study. They were excluded if they had evidence of possible helminth-induced pathology (severe anemia, clinically apparent liver disease, or bloody diarrhea), if the pregnancy was abnormal, or if they had already enrolled during a previous pregnancy. The mothers and infants underwent intensive surveillance during the first year of infant life. Blood samples were taken and stored from both mother and cord blood around the time of birth. Samples, including whole blood, were subsequently obtained from the child annually on or around their birthday (2 to 5 ml depending on the age). The child’s samples were subsequently divided into plasma and red cell pellets as described in more detail below (*23*). Infants were included in the present study if (i) receipt of three doses of DTwP (a combination of diphtheria and tetanus toxoids and inactivated ‘whole’-cell pertussis organism vaccine)/Hib (*Haemophilus influenzae* type b)/HBV (hepatitis B virus) (at approximately 6, 10, and 14 weeks of age) and one dose of measles vaccine (at approximately 9 months of age) could be confirmed from their vaccination records, (ii) DNA could be extracted from stored red cell pellets, and (iii) plasma samples were available from the 12-month age point of sampling. Ethical approval was provided locally by the Uganda Virus Research Institute (reference GC/127/12/07/32) and Uganda National Council for Science and Technology (MV625), and in the United Kingdom by the London School of Hygiene and Tropical Medicine (A340) and Oxford Tropical Research (39-12 and 42-14) Ethics Committees.

#### South Africa: The Soweto Vaccine Response Study

Six-month old infants born in Chris Hani Baragwanath Hospital living in the Soweto region of Johannesburg, South Africa, were identified from screening logs and databases of participants involved in clinical vaccine trials (*24*) coordinated by the Respiratory and Meningeal Pathogens Unit (www.rmpru.com/). Mothers of the infants were approached if the infants had received all of their vaccines up to 6 months of age (DTaP/Hib/HBV at approximately 4, 8, and 12 weeks of age). After receiving information about the study, the mothers were consented in accordance with ethical approval from the University of Witwatersrand Human Research Ethics Committee (reference M130714) and the Oxford Tropical Research Ethics Committee (1042-13 and 42-14). The infants were sampled prospectively at 6 months of age and at 12 months after receipt of measles vaccine at 9 months. Single whole-blood samples were collected and prepared using a similar protocol to that used in Entebbe to extract DNA from cell pellets and plasma for antibody assays.

#### Burkina Faso: The VAC050 ME-TRAP malaria vaccine trial

Infants between the ages of 6 and 18 months living in the Banfora region of Burkina Faso were recruited into a phase 1/2b clinical trial to test the safety, immunogenicity, and efficacy of an experimental heterologous viral-vectored prime-boost liver-stage malaria vaccine (*25*). These infants were all expected to receive their EPI (Expanded Programme on Immunization) vaccines as part of the usual national schedule (4, 8, and 12 weeks of age). Infants were precluded from participating in the trial if they were found to have clinical or hematological (venous hemoglobin <8 g/dl) evidence of severe anemia, history of allergic or neurological disease, or malnutrition. Of a total of 730 infants who were recruited into the study, samples suitable for extraction of DNA were collected and stored from 400 infants (350 vaccine recipients and 50 recipients of a control rabies vaccine). Samples of plasma were available from the infants at multiple time points following receipt of the experimental vaccine. Samples from individuals taken at time points as close to the 12-month age as possible were prioritized for EPI vaccine response measurements. The infants underwent intensive clinical history and examination during screening and follow-up. The mothers of the participating infants provided consent for their children to be enrolled in the clinical trial and for subsequent genetic studies to be undertaken for all vaccines received in accordance with ethical approval from the Ministere de la Recherche Scientifique et de l’Innovation in Burkina Faso (reference 2014-12-151) and the Oxford Tropical Research Ethics Committee (reference 41-12).

### Other study populations

#### The Gambia: West Kiang study

All children aged 2 to 6 years (except those with chronic illness) were recruited from 10 rural villages in the West Kiang region of The Gambia during the malaria season (July to August 2001) (*26*). Ethnic groups were Mandinka (9 villages, 700 children) and Fulani (1 village, 80 children). All children had a clinical examination, anthropometric measurements, and a 3-day course of mebendazole for potential hookworm infection. A blood sample was collected for complete blood count, malaria slide, iron status assays, haptoglobin concentrations, ACT (a marker of inflammation), and DNA extraction. Children with a temperature >37.5°C had a malaria blood film, appropriate clinical treatment, and a blood sample 2 weeks later after recovery from illness. Parental written informed consent was obtained for all study participants. Ethical approval was provided by the Gambian government/Medical Research Council Ethics Committee (874/830).

#### Kenya: Ngerenya community-based cohort

This is an ongoing rolling cohort evaluating malaria immunity in children at the KEMRI-Wellcome Trust Research Programme as described elsewhere (*27*). Briefly, children were followed-up to a maximum age of 13 years with annual cross-sectional bleeds. The follow-ups involved weekly visits to assess for fever and temperature. If temperature was above 37.5°C, a malaria blood film was taken, and if positive, treatment was provided. Iron and inflammatory biomarkers and red cell indices were measured from a single cross-sectional bleed based on the availability of plasma samples archived at −80°C.

Other data used in this study are described elsewhere including case-control analyses of severe malaria in children from Kenya, Malawi, The Gambia, and Ghana recruited and analyzed as part of MalariaGEN (www.malariagen.net/data/genome-wide-study-resistance-severe-malaria-eleven-populations) (*8*) and the Kenyan bacteremia study (*9*). The individuals involved in coordinating, recruiting, and managing the samples and data from the Kenyan bacteremia study are listed in Appendix A.

### Laboratory methods

#### Blood sampling and preparation

Whole blood was sampled into vacutainer tubes (BD, Becton Dickinson and Company, New Jersey, USA) containing ethylenediaminetetraacetic acid (for the Ugandan and South African studies) or lithium heparin (Burkinabe, Kilifi, and West Kiang) as an anticoagulant in all five populations. Following centrifugation, the samples were separated into their constituent parts (plasma, buffy coat, and red cell/erythrocyte layers) and stored at −80°C until downstream analysis in batches. DNA was extracted from the erythrocyte layer in the Ugandan study and from the buffy coat in the South African, Burkinabe, Kilifi, and West Kiang studies. DNA from all cohorts was extracted from the relevant samples using the Qiagen QIAamp DNA Mini or Midi Kits (Qiagen, Hilden, Germany) using recommended protocols. Whole blood was also sampled into serum separator tubes (SST; BD, New Jersey USA) in the Ugandan study, and serum was isolated and stored according to the recommended protocols.

#### Laboratory assays

The assayed biomarkers of iron included plasma ferritin [Chemiluminescent Microparticle Immunoassay (CMI), Abbott Architect, USA], iron (MULTIGENT iron calorimetric assay, Abbott Architect, USA), transferrin (CMI, Abbott Architect, USA), and zinc protoporphyrin (Aviv Biomedical Hematoflurometer within 24 hours of collection). Hepcidin was assayed by using the DRG Hepcidin 25 (bioactive) high-sensitive ELISA (enzyme-linked immunosorbent assay) Kit (DRG International, USA) in Uganda, South Africa, Burkinabe, and Kilifi, or by using the Hepcidin-25 (human) EIA (enzyme immunoassay) Kit (Bachem, Switzerland) in West Kiang. Soluble transferrin receptor was assayed by using the Human Soluble Transferrin Receptors (sTfR) ELISA (BioVendor, Czech Republic) in Uganda, South Africa, Burkina Faso, and Kilifi, or by the Quantikine sTfR ELISA Kit (R&D Systems, USA) in West Kiang. Since biomarkers of iron are influenced by inflammation, C-reactive protein (MULTIGENT CRP Vario assay, Abbott Architect, USA in Uganda, South Africa, Burkina Faso, and Kilifi) or ACT (Immunoturbidimetry, Cobas Mira Plus Bio-analyzer, Roche in West Kiang) was assayed to adjust for inflammation (*28*). The red cell parameters including hemoglobin were analyzed using the Medonic CA 530 Oden 16 hemoglobinometer. Haptoglobin concentrations were analyzed using the Tina Quant Haptoglobin Kit, Roche Diagnostics, Cobas Bio centrifugal analyzer (West Kiang).

#### Genotyping and SNP quality control

The single single-nucleotide polymorphism (SNP) encoding the *FPN* Q248H mutation was directly genotyped in all populations, except Ghana, which is described further below. The VaccGene populations were all genotyped using the Illumina HumanOmni 2.5 M-8 (“octo”) BeadChip array version 1.1 (Illumina Inc., San Diego, USA), performed by the Genotyping Core facilities at the Wellcome Trust Sanger Institute. Genomic DNA underwent whole-genome amplification and fragmentation before hybridization to locus-specific oligonucleotides bound to 3-μm-diameter silica beads. Fragments were extended by single-base extension to interrogate the variant by incorporating a labeled nucleotide enabling a two-color detection (Illumina). Genotypes were called from intensities using two clustering algorithms (Illuminus and GenCall) in GenomeStudio (Illumina Inc., San Diego, USA) incorporating data from proprietary predetermined genotypes.

SNP quality control (QC) was performed separately for each cohort using identical steps and using SNPs mapped to Human Genome Build 37. Low-quality variants that mapped to multiple regions within the human genome or did not map to any region were removed. Samples with a call rate of less than 97% and heterozygosity greater than 3 SDs around the mean were filtered sequentially. Sex check was performed in PLINK (v1.7) (http://zzz.bwh.harvard.edu/plink/) using default *F* values of <0.2 for males and >0.8 for females. Samples with discordance between reported and genetic sex were removed. Genetic variant filtering was performed across the remaining samples, and sites called in <97% samples were removed from each population. Identity by descent (IBD) was measured within each population. Only samples with IBD >0.9 not known to be twins were removed using an algorithm that removed the sample from the pair with the lower variant call rate. Sites in the Hardy-Weinberg disequilibrium (*P* < 10^−8^) were also excluded from future analysis in all individuals, calculated using individuals with IBD <0.05 (hereafter designated “founders”). Following the above quality control steps, principal component analysis (PCA) was performed in EIGENSOFT v4.2 (www.hsph.harvard.edu/alkes-price/software/) for each population and combined with populations from the 1000 Genomes (*13*). PCA was carried out after linkage disequilibrium pruning to a threshold of *r*^2^ = 0.5 using a sliding window approach with a window size of 50 SNPs sliding 5 SNPs sequentially. Regions of long-range linkage disequilibrium were removed from the analysis. Individuals with values of the first 10 principal components >6 SDs around the mean of other samples in each population were removed. The variant encoding the *FPN* Q248H mutation was retained in all datasets following the stringent quality control processes described: rs11568350 (www.ncbi.nlm.nih.gov/projects/SNP/snp_ref.cgi?rs=11568350).

For the Kenyan and Gambian community-based cohorts, the Q248H (rs11568350) mutation was directly typed using Agena Bioscience SEQUENOM matrix-assisted laser desorption/ionization–time-of-flight (MALDI-TOF) mass spectrometry on the iPLEX platform (*29*). Briefly genomic DNA was whole genome amplified using primer extension preamplification (*30*) before genotyping as per the manufacturer’s instructions for the iPLEX platform. Assay primers for Q248H were designed using the SpectroDESIGNER(R) primer design software and ordered from Metabion (www.metabion.com). Genotypes were called and curated by inspecting cluster plots using the SpectroTYPER software. The Q248H SNP had a call rate of 100% and was in the Hardy-Weinberg equilibrium (Pearson chi-square *P* = 0.32).

Details of genotyping of the severe malaria cases, other than Ghanaian trio probands, and bacteremia cases and the 1000 Genomes project are described elsewhere (*8*, *9*, *13*). We selected the Q248H SNP from the genotyped data.

To obtain genotypes for Q248H in the Ghanaian trios, we downloaded the genotype array data from the European Genome-Phenome Archive (dataset ID EGAD00000000020) and implemented sample and SNP QC as suggested in the documentation. We used the strand file produced by W. Rayner (www.well.ox.ac.uk/~wrayner/strand/) for the Illumina Omni 650Y array to realign SNPs to Human Genome Build 37. We then used SHAPEIT2 to statistically phase genotypes genome-wide, based on the family trio structure and patterns of linkage disequilibrium. We imputed genotypes into the 1000 Genomes Phase 3 reference panel using IMPUTE2 (*31*). Q248H was imputed with high confidence (IMPUTE info = 0.99). We extracted Q248H from the imputed data for further analysis.

### *P. falciparum* in vitro growth assay

In vitro parasite growth was assessed in fresh, washed RBCs for 96 hours using the *P. falciparum* strain FCR3-FMG (MR4, MRA-736) as previously described (*32*). Other laboratory strains and field isolates have previously been shown to yield similar results (*32*, *33*). Briefly, synchronized parasite cultures were seeded as rings into RBCs from study participants in triplicate into 96-well plates at 0.5% initial parasitized RBCs in 20 × 10^6^ RBC total per well (1% hematocrit) and maintained for 96 hours. Identical plates were set up in parallel for assaying precise 0-hour parasitemia in each blood sample. Growth rates represent the final 96-hour parasitemia divided by the initial 0-hour parasitemia, analyzed by flow cytometry staining for parasitized RBCs using DNA dye SYBR Green I (Invitrogen).

RBCs used were obtained from Gambian children and pregnant women enrolled in randomized trials of hepcidin-guided iron supplementation (*10*, *11*). Children (6 to 27 months) were screened as anemic (hemoglobin <11 g/dl) at enrollment but otherwise healthy, and blood was collected at days 0, 49, and 84 during 12 weeks of iron supplementation. Pregnant women (18 to 45 years) were estimated 14 to 22 weeks’ gestation at enrollment, and blood was collected at days 0, 14, 49, and 84 during 12 weeks of iron supplementation. Growth rate data from all blood collection time points were analyzed in this study. Children contributed 1 to 3 data points and women 1 to 4 data points. RBCs from healthy, iron-replete adult donors of normal hemoglobin genotype and G6PD (glucose-6-phosphate dehydrogenase) status not undergoing iron supplementation served as controls for interassay variability. Growth rates in the subjects’ RBCs were normalized to that in control RBCs assayed simultaneously. *FPN* genotyping for the Q248H polymorphism was performed using DNA extracted from the subjects’ blood (QIAamp DNA Blood Mini Kit, Qiagen) and Thermo Fisher’s TaqMan SNP assay (Thermo Fisher Scientific 4351379, protocol-MAN0009593). Statistical analyses were performed in DataDesk 7.0.2 (Data Description Inc., Ithaca, NY).

### Definitions

Iron deficiency was defined as plasma ferritin <12 or <30 μg/liter in the presence of inflammation (defined as C-reactive protein >5 mg/liter or ACT >0.6 g/liter) in children <5 years, or <15 μg/liter in children ≥5 years (*28*). Anemia was defined as hemoglobin <11 g/dl (*34*). Iron deficiency anemia was defined as presence of iron deficiency and anemia (*34*) . Malaria parasitemia was defined as asexual *P. falciparum* parasite positive. Severe malaria was defined as positive for *P. falciparum* parasites in the blood film and clinical features of severe malaria as defined by World Health Organization criteria (*8*). Cerebral malaria was defined as a case of severe malaria with a Blantyre coma score of <3, and severe malarial anemia was defined as a case of severe malaria with a hemoglobin level of <5 g/dl or a hematocrit level of <15% (*8*). Bacteremia was defined as a positive pathogenic blood culture.

### Statistical analysis

All statistical analyses of community-based cohorts were conducted using STATA 13.1 (StataCorp, College Station, TX). Individual iron markers were normalized by log transformation. Proportions were compared between groups by chi-square or Fisher’s exact test where appropriate, and continuous variables by Student’s *t* test or Mann-Whitney *U* test. Linear and logistic regression models were adjusted for age, sex, and cohort. Analyses of malaria and bacteremia cohorts were undertaken using logistic regression in R and multinomial regression in SNPTEST (version 2.5), respectively. For the malaria case-control analysis undertaken for trios in Ghana, a transmission disequilibrium test was undertaken using the “trio” package in R. Meta-analyses were performed using the “metan” package in STATA.

Estimates of heterozygosity at the DNA variant encoding the *FPN* Q248H variant (using Fisher’s exact test) and *F*_ST_ (using the Weir and Cockerham method) were calculated using the “diveRsity” package available in R (*35*). To investigate the relationship between historical rates of malaria transmission, the minor allele frequencies of this variant within each population were compared with estimates of *P. falciparum* transmission rates estimated between 1900 and 1959 using data recently made publicly available (*14*). The maximal transmission rate for any geocoded region between 1900 and 1959 was used to create a composite measure that was expected to contribute most to natural selection, if present. Regions, including those where the populations were recruited from, were included in the analysis. Shapefiles were loaded and transformed using the “raster” (http://rspatial.org/), “sp” (https://edzer.github.io/sp/), and “rgdal” (www.gdal.org/) packages and then plotted and fortified using “ggplot2” (https://ggplot2.tidyverse.org/) in R. As a comparison for more modern data, an equivalent method was used using data from the Malaria Atlas Project (MAP) spanning the years 2000–2015. GPS coordinates for study sites were uploaded to MAP (https://map.ox.ac.uk/), and the *P. falciparum* parasite rates in 2- to 10-year-olds in Africa were plotted against rs11568350-derived allele frequency and correlations were compared within years using Spearman rank tests of correlation.

## Supplementary Material

http://advances.sciencemag.org/cgi/content/full/5/9/eaaw0109/DC1

Download PDF

The ferroportin Q248H mutation protects from anemia, but not malaria or bacteremia

## SUPPLEMENTARY MATERIALS

Supplementary material for this article is available at http://advances.sciencemag.org/cgi/content/full/5/9/eaaw0109/DC1 Fig. S1. A meta-analysis of studies investigating the relationship between the *FPN* Q248H mutation and hemoglobin levels. Fig. S2. Meta-analysis of associations of the *FPN* Q248H mutation with anemia, hemoglobin, and iron status across the study populations. Fig. S3. Forest plots of the effect of the *FPN* Q248H mutation on hemoglobin and measures of iron status. Fig. S4. Correlation between *P. falciparum* prevalence/rate and the derived adenine allele encoding the Q248H mutation. Table S1. Summary of studies examining the relationship between the *FPN* Q248H mutation and hemoglobin, ferritin, and C-reactive protein. Table S2. Characteristics of participants by study cohort and *FPN* Q248H mutation. Table S3. Estimates of the effect of Q248H heterozygotes and homozygotes on iron status and anemia. Table S4. Estimates of the effect of Q248H heterozygotes and homozygotes on severe malaria and bacteremia status. Table S5. Deviation from Hardy-Weinberg equilibrium for the variant causing the *FPN* Q248H mutation. Table S6. Rare alleles present in populations included in the 1000 Genomes Phase 3. Appendix A References (*36*–*40*)
