# Supplementary material for: The ferroportin Q248H mutation protects from anemia, but not malaria or bacteremia
Source: Sci Adv. 2019 Sep 4;5(9):eaaw0109. doi: 10.1126/sciadv.aaw0109 (PMC6726445; doi:10.1126/sciadv.aaw0109)
Supplement: http://advances.sciencemag.org/cgi/content/full/5/9/eaaw0109/DC1 [file supp_5_9_eaaw0109__index.html]

Science Advances | Science AdvancesAAASSearchScience AdvancesMenu

## Supplementary Materials

**This PDF file includes:**

- Fig. S1. A meta-analysis of studies investigating the relationship between the *FPN* Q248H mutation and hemoglobin levels.
- Fig. S2. Meta-analysis of associations of the *FPN* Q248H mutation with anemia, hemoglobin, and iron status across the study populations.
- Fig. S3. Forest plots of the effect of the *FPN* Q248H mutation on hemoglobin and measures of iron status.
- Fig. S4. Correlation between *P. falciparum* prevalence/rate and the derived adenine allele encoding the Q248H mutation.
- Table S1. Summary of studies examining the relationship between the *FPN* Q248H mutation and hemoglobin, ferritin, and C-reactive protein.
- Table S2. Characteristics of participants by study cohort and *FPN* Q248H mutation.
- Table S3. Estimates of the effect of Q248H heterozygotes and homozygotes on iron status and anemia.
- Table S4. Estimates of the effect of Q248H heterozygotes and homozygotes on severe malaria and bacteremia status.
- Table S5. Deviation from Hardy-Weinberg equilibrium for the variant causing the *FPN* Q248H mutation.
- Table S6. Rare alleles present in populations included in the 1000 Genomes Phase 3.
- Appendix A
- References (*36*–*40*)

Download PDF

**Files in this Data Supplement:**

- Adobe PDF - aaw0109\_SM.pdf
