# Supplementary material for: The ferroportin Q248H mutation protects from anemia, but not malaria or bacteremia
Source: Sci Adv. 2019 Sep 4;5(9):eaaw0109. doi: 10.1126/sciadv.aaw0109 (PMC6726445; doi:10.1126/sciadv.aaw0109)
Supplement: Download PDF [file aaw0109_SM.pdf]

Science Advances
AAAS

advances.sciencemag.org/cgi/content/full/5/9/eaaw0109/DC1

Supplementary Materials for

The ferroportin Q248H mutation protects from anemia, but not malaria or bacteremia

John Muthii Muriuki, Alexander J. Mentzer*, Gavin Band, James J. Gilchrist, Tommy Carstensen, Swaib A. Lule, Morgan M. Goheen, Fatou Joof, Wandia Kimita, Reagan Mogire, Clare L. Cutland, Amidou Diarra, Anna Rautanen, Cristina Pomilla, Deepti Gurdasani, Kirk Rockett, Neema Mturi, Francis M. Ndungu, J. Anthony G. Scott, Sodiomon B. Sirima, Alireza Morovat, Andrew M. Prentice, Shabir A. Madhi, Emily L. Webb, Alison M. Elliott, Philip Bejon, Manjinder S. Sandhu, Adrian V. S. Hill, Dominic P. Kwiatkowski, Thomas N. Williams, Carla Cerami, Sarah H. Atkinson*

*Corresponding author. Email: alexander.mentzer@ndm.ox.ac.uk (A.J.M.); satkinson@kemri-wellcome.org (S.H.A.)

Published 4 September 2019, *Sci. Adv.* **5**, eaaw0109 (2019)
DOI: 10.1126/sciadv.aaw0109

This PDF file includes:

Fig. S1. A meta-analysis of studies investigating the relationship between the *FPN* Q248H mutation and hemoglobin levels.
Fig. S2. Meta-analysis of associations of the *FPN* Q248H mutation with anemia, hemoglobin, and iron status across the study populations.
Fig. S3. Forest plots of the effect of the *FPN* Q248H mutation on hemoglobin and measures of iron status.
Fig. S4. Correlation between *P. falciparum* prevalence/rate and the derived adenine allele encoding the Q248H mutation.
Table S1. Summary of studies examining the relationship between the *FPN* Q248H mutation and hemoglobin, ferritin, and C-reactive protein.
Table S2. Characteristics of participants by study cohort and *FPN* Q248H mutation.
Table S3. Estimates of the effect of Q248H heterozygotes and homozygotes on iron status and anemia.
Table S4. Estimates of the effect of Q248H heterozygotes and homozygotes on severe malaria and bacteremia status.
Table S5. Deviation from Hardy-Weinberg equilibrium for the variant causing the *FPN* Q248H mutation.
Table S6. Rare alleles present in populations included in the 1000 Genomes Phase 3.
Appendix A
References (*36*–*40*)

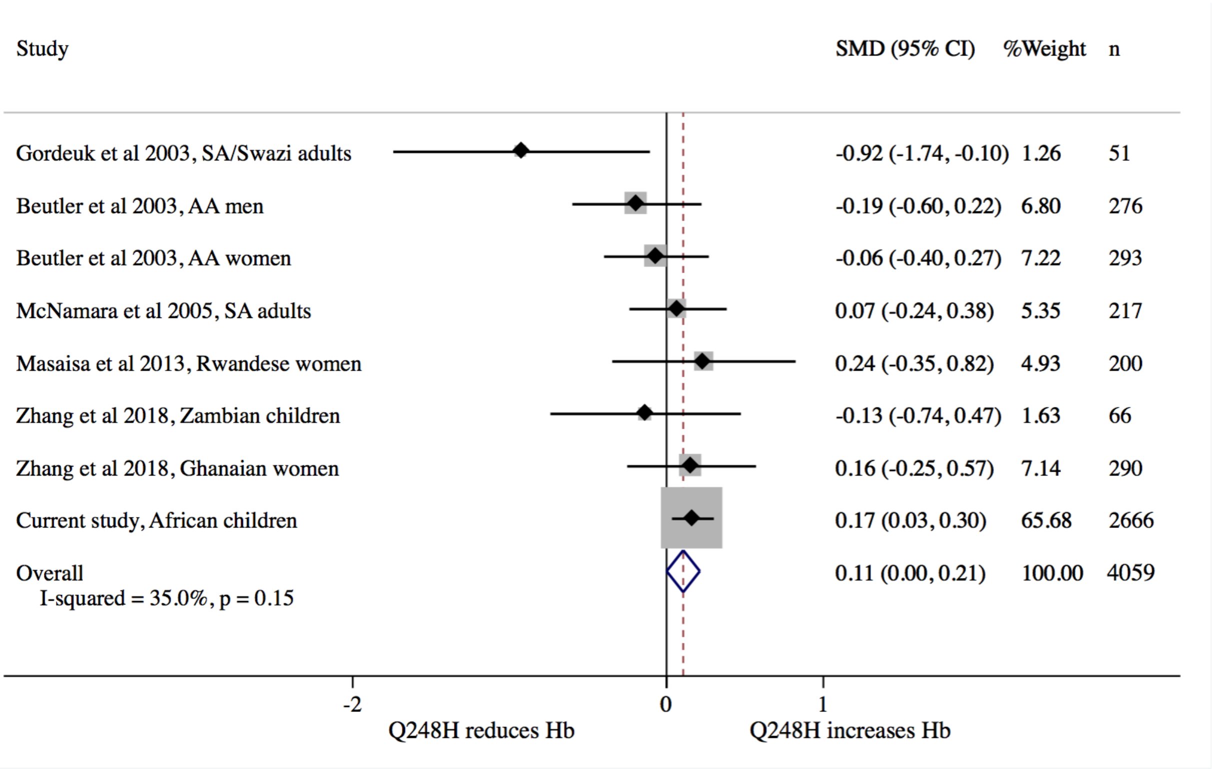

Fig. S1. A meta-analysis of studies investigating the relationship between the *FPN* Q248H mutation and hemoglobin levels. The current study included Ugandan, Gambian, Burkinabe, and Kenyan children. Table S1 gives more details of the studies included in the meta-analysis. The grey boxes indicate sample size. SA, South Africa; AA, African-American; SMD, standardized mean difference.

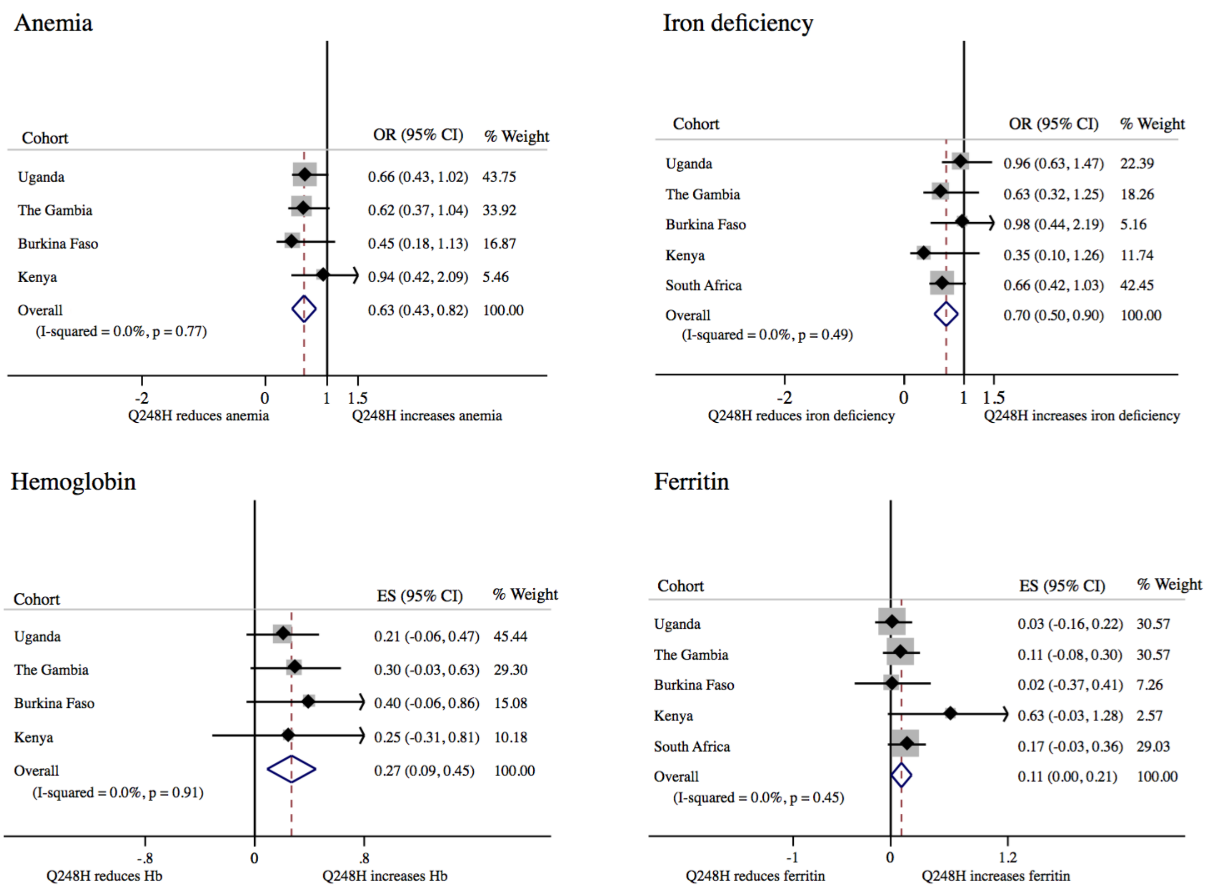

Fig. S2. Meta-analysis of associations of the *FPN* Q248H mutation with anemia, hemoglobin, and iron status across the study populations. Labels indicate odds ratios or estimates and 95% confidence intervals. Ferritin was normalized by log-transformation. Hemoglobin measurements were not available for the South African cohort. Regression models were adjusted for age and sex. OR, Odds Ratio; ES, Effect Size.

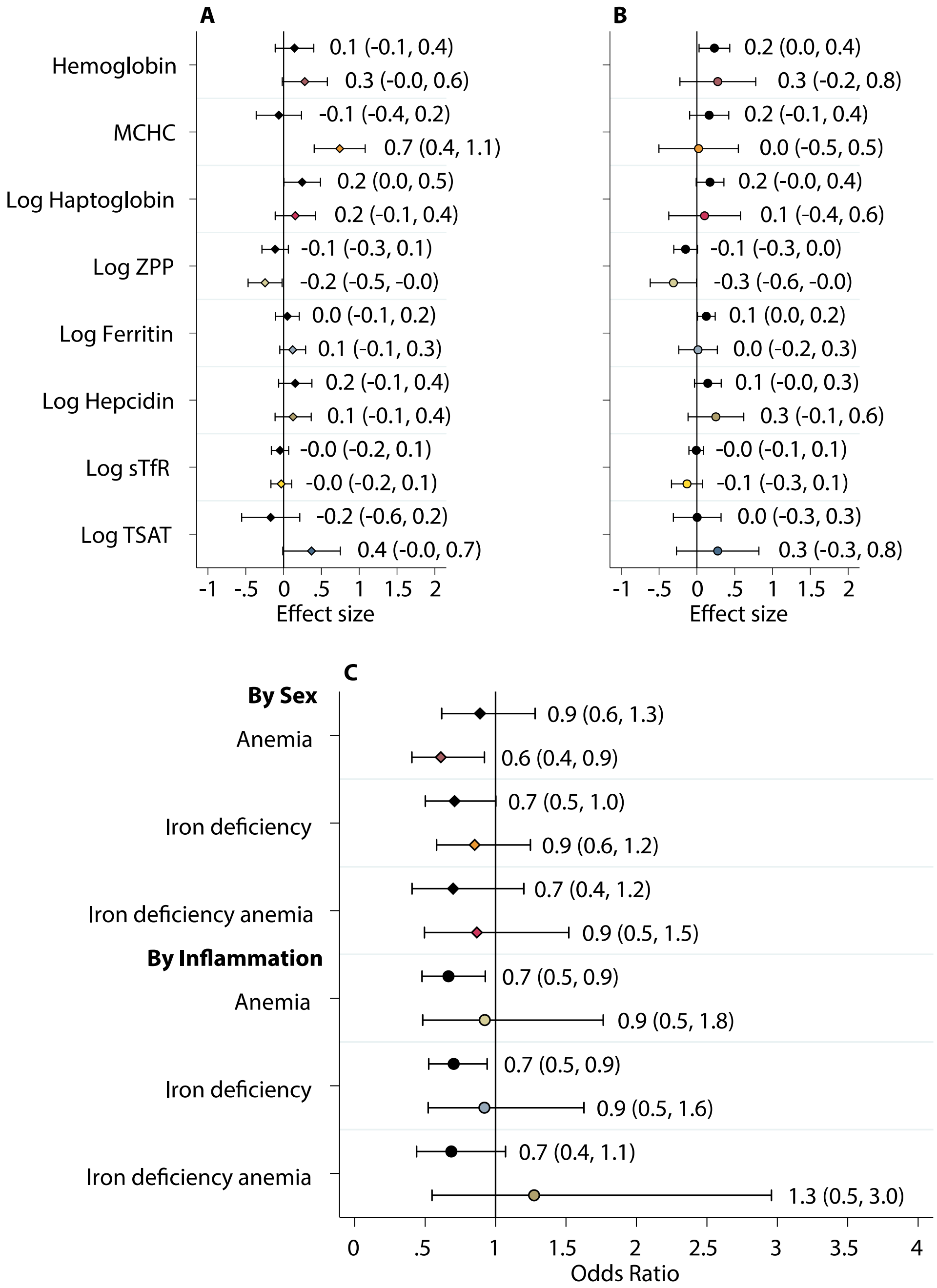
A
Hemoglobin
MCHC
Log Haptoglobin
Log ZPP
Log Ferritin
Log Hepcidin
Log sTfR
Log TSAT
0.1 (-0.1, 0.4)
0.3 (-0.0, 0.6)
-0.1 (-0.4, 0.2)
0.7 (0.4, 1.1)
0.2 (0.0, 0.5)
0.2 (-0.1, 0.4)
-0.1 (-0.3, 0.1)
-0.2 (-0.5, -0.0)
0.0 (-0.1, 0.2)
0.1 (-0.1, 0.3)
0.2 (-0.1, 0.4)
0.1 (-0.1, 0.4)
-0.0 (-0.2, 0.1)
-0.0 (-0.2, 0.1)
-0.2 (-0.6, 0.2)
0.4 (-0.0, 0.7)
-1 -.5 0 .5 1 1.5 2
Effect size
B
0.2 (0.0, 0.4)
0.3 (-0.2, 0.8)
0.2 (-0.1, 0.4)
0.0 (-0.5, 0.5)
0.2 (-0.0, 0.4)
0.1 (-0.4, 0.6)
-0.1 (-0.3, 0.0)
-0.3 (-0.6, -0.0)
0.1 (0.0, 0.2)
0.0 (-0.2, 0.3)
0.1 (-0.0, 0.3)
0.3 (-0.1, 0.6)
-0.0 (-0.1, 0.1)
-0.1 (-0.3, 0.1)
0.0 (-0.3, 0.3)
0.3 (-0.3, 0.8)
-1 -.5 0 .5 1 1.5 2
Effect size
C
By Sex
Anemia
Iron deficiency
Iron deficiency anemia
By Inflammation
Anemia
Iron deficiency
Iron deficiency anemia
0.9 (0.6, 1.3)
0.6 (0.4, 0.9)
0.7 (0.5, 1.0)
0.9 (0.6, 1.2)
0.7 (0.4, 1.2)
0.9 (0.5, 1.5)
0.7 (0.5, 0.9)
0.9 (0.5, 1.8)
0.7 (0.5, 0.9)
0.9 (0.5, 1.6)
0.7 (0.4, 1.1)
1.3 (0.5, 3.0)
0 .5 1 1.5 2 2.5 3 3.5 4
Odds Ratio

Fig. S3. Forest plots of the effect of the *FPN* Q248H mutation on hemoglobin and measures of iron status A) stratified by sex, **B**) stratified by inflammation, and **C)** of *FPN* Q248H on anemia, iron deficiency, and iron deficiency anemia stratified by sex and inflammation. Solid diamond markers indicate males; hollow diamond, females; solid circle, no inflammation; hollow circle, those with inflammation. Markers indicate the estimate (β) or odds ratio, while error bars indicate 95% confidence intervals and are labeled respectively. Regression models were adjusted for age and cohort for the analyses stratified by gender and for age, cohort and gender for the analyses stratified by inflammation. No significant interactions were observed between the mutation and either gender or inflammation in predicting iron status.

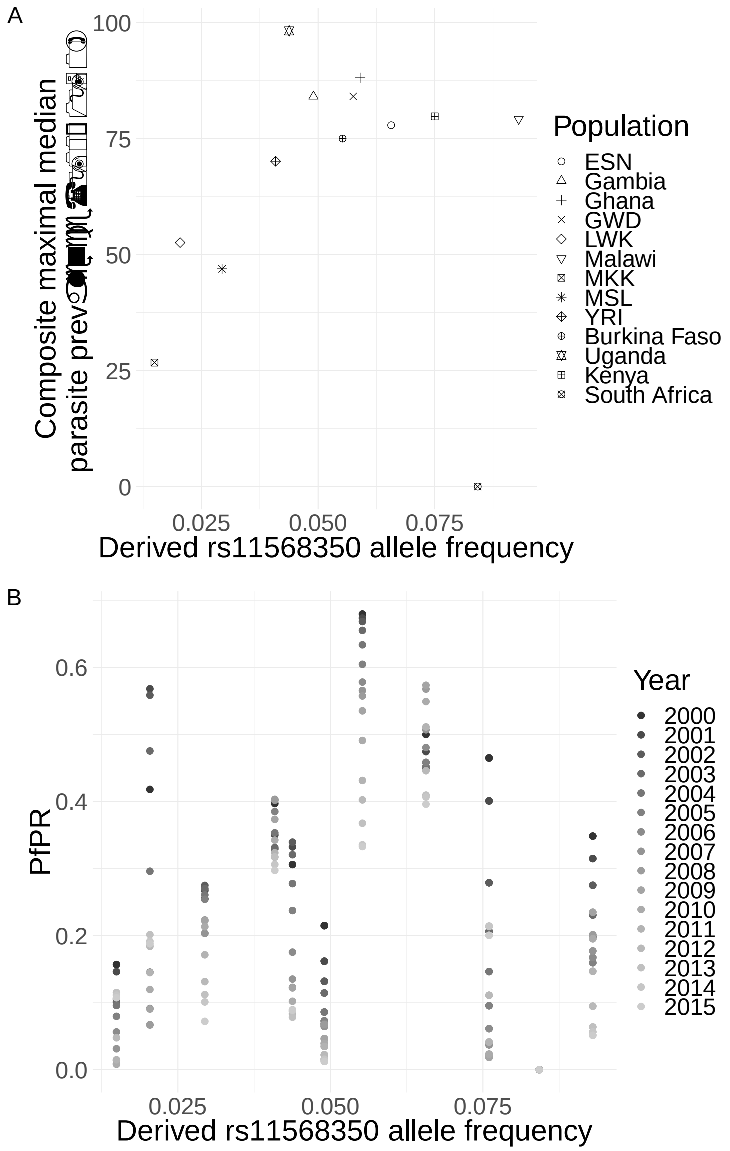

Fig. S4. Correlation between *P. falciparum* prevalence/rate and the derived adenine allele encoding the Q248H mutation. A) shows the correlation with *P. falciparum* maximal median parasite prevalence between 1900 and 1959 across 13 tested African populations. Each point represents one population with the *FPN* Q248H derived allele frequency as specified on the x-axis with parasite intensity defined on the y-axis using parasite prevalence available data generated from Snow *et al* 2017 (*14*). Exclusion of South African individuals as outliers resulted in non-significant increase in correlation (Rho 0.57; $P = 0.06$). **B)** shows correlation with *P. falciparum* parasite rate (*Pf*PR) between the years of 2000-2015. Each column of points represents either a single population, or a set of populations with the ferroportin Q248H derived allele frequency specified on the x-axis compared against *Pf*PR on the y-axis colored by year of available data generated from the Malaria Atlas Project.

Table S1. Summary of studies examining the relationship between the *FPN* Q248H mutation and hemoglobin, ferritin, and C-reactive protein.

Study	Country	Age	Sample size	Q248H, n	WT, n	Summary statistic	Q248H	Wild Type
**Hemoglobin, g/dl**								
Gordeuk et al 2003[*] (*15*)	South Africa and Swaziland	Adults	51	7	44	Mean (±SD)	12.5 (±1.3)	13.7 (±1.3)
Beutler et al 2003[†], males (*36*)	USA - African Americans	Adults	276	25	251	Mean (±SD)	14.2 (±1.5)	14.5 (±1.6)
Beutler et al 2003, females (*36*)	USA - African Americans	Adults	293	40	253	Mean (±SD)	12.5 (±1.3)	12.6 (±1.6)
McNamara et al 2005[†] (*37*)	South Africa	Adults	217	53	164	Mean (±SD)	13.6 (±1.5)	13.5 (±1.3)
Masaisa et al 2012[‡] (*19*)	Rwanda	Women	200	12	188	Mean (±SD)	13.1 (±3.4)	12.4 (±2.9)
Zhang et al 2018 (*3*)	Zambia	Children	66	13	53	Mean (±SD)	9.2 (±2.5)	9.5 (±2.2)
Zhang et al 2018 (*3*)	Ghana	Women	290	25	265	Mean (±SD)	11.5 (±1.5)	11.2 (±1.9)
Kasvosve et al 2010 (*38*)	Zimbabwe	Children	59	13	46	Median (IQR)	11.2 (10.6, 12.0)	10.9 (10.3, 11.5)
Current study[§]	Across Africa	Children	2666	237	2429	Mean (±SD)	10.8 (±1.1)	10.6 (±1.2)
**Ferritin, ng/ml or µg/L**								
Gordeuk et al 2003 (*15*)	South Africa and Swaziland	Adults	51	7	44	Geometric mean (95% CI)	63.0 (40.0, 100.0)	35.0 (29.0, 42.0)
Beutler et al 2003, males (*36*)	USA - African Americans	Adults	276	25	251	Geometric mean (95% CI)	141.8 (93.9, 214.4)	158.4 (141.3, 177.5)
Beutler et al 2003, females (*36*)	USA - African Americans	Adults	293	40	253	Geometric mean (95% CI)	62.9 (40.5, 97.9)	51.2 (44.4, 59.0)
Kasvosve et al 2005 (*16*)	Zimbabwe	Children	195	38	157	Geometric mean (95% CI)	32.0 (27.0, 39.0)	21.0 (19.0, 23.0)
McNamara et al 2005 (*37*)	South Africa	Adults	217	53	164	Geometric mean (95% CI)	163.0 (138.0, 193.0)	131.0 (119.0, 144.0)
Katchunga et al 2013, controls (*39*)	DRC	Adults	86	7	79	Geometric mean (95% CI)	105.3 (92.3, 186.7)	128.3 (65.1, 228.7)
Katchunga et al 2013, diabetic (*39*)	DRC	Adults	179	25	154	Geometric mean (95% CI)	214.4 (121.2, 309.1)	183.6 (97.1, 292.6)
Nekhai et al 2013[**] (*4*)	USA	Adults	78	9	69	Geometric mean (95% CI)	88.0 (29.0, 273.0)	211.0 (74.0, 602.0)

Kasvosve et al 2010 (*38*)	Zimbabwe	Children	64	14	50	Median (IQR)	17.0 (11.0, 29.0)	20.0 (10.0, 33.0)
Masaisa et al 2012 (*19*)	Rwanda	Women	200	12	188	Median (IQR)	295.0 (179.0, 363.0)	74.9 (47.2, 118.0)
Kasvosve et al 2015[††], males (*18*)	Botswana	Adults	84	14	70	Median (IQR)	127.4 (97.2, 192.5)	79.7 (50.6, 137.0)
Kasvosve et al 2015, females (*18*)	Botswana	Adults	74	12	62	Median (IQR)	47.4 (22.2, 83.8)	38.5 (24.1, 68.1)
Cikomola et al 2017 (*40*)	DRC	Adults	42	15	27	Median (IQR)	211.0 (142.0, 476.0)	228.0 (115.0, 540.0)
Current study	Across Africa	Children	3065	325	2740	Geometric mean (95% CI)	20.8 (18.8, 23.0)	20.0 (19.3, 20.8)
**C-reactive protein, mg/L**								
Masaisa et al 2012 (*19*)	Rwanda	Women	200	12	188	Mean (±SD)	5.2 (±2.0)	4.8 (±1.2)
McNamara et al 2005 (*37*)	South Africa	Adults	217	53	164	Geometric mean (95% CI)	4.5 (4.0, 5.1)	2.7 (2.5, 2.9)
Kasvosve et al 2005 (*16*)	Zimbabwe	Children	195	38	157	Median (IQR)	0.8 (0.3, 2.8)	0.6 (0.1, 2.5)
Kasvosve et al 2010 (*38*)	Zimbabwe	Children	64	14	50	Median (IQR)	1.0 (0.3, 2.0)	0.5 (0.1, 2.0)
Current study	Across Africa	Children	2403	263	2140	Geometric mean (95% CI)	1.1 (0.9, 1.3)	1.4 (1.3, 1.5)

[*] We used community controls. We calculated standard deviation from the provided standard error
[†] Participants had iron overload. We calculated standard deviation from the provided standard error.
[‡] HIV positive women.
[§] The current study includes data from Kenya, Uganda, South Africa, Burkina Faso and The Gambia.
[**] Patients with sickle cell anemia and transfused <5 units of blood.
[††] Analyzed data for those with serum ferritin ≥10ng/mL and ≤200ng/mL.
CI, confidence interval; DRC, Democratic Republic of Congo; IQR, interquartile range; SD, standard deviation; USA, United States of America.

Table S2. Characteristics of participants by study cohort and *FPN* Q248H mutation.

	Uganda			The Gambia			South Africa			Burkina Faso			Kenya		
	Q248H (n=117)	WT (n=1248)	P	Q248H (n=71)	WT (n=675)	P	Q248H (n=102)	WT (n=518)	P	Q248H (n=34)	WT (n=313)	P	Q248H (n=31)	WT (n=265)	P
Median age in years (IQR)	2.0 (2.0-3.0)	2.0 (2.0-3.0)	0.90	3.8 (2.9-4.5)	3.8 (2.9-4.9)	0.56	1.0 (1.0-1.0)	1.0 (1.0-1.0)	0.35	1.9 (1.6-2.1)	1.9 (1.7-2.2)	0.32	4.2 (2.1-6.1)	4.1 (2.3-6.0)	0.87
Females, n (%)	49 (41.9)	622 (49.8)	0.10	32 (45.1)	315 (46.7)	0.80	46 (45.1)	253 (48.8)	0.49	18 (52.9)	154 (49.2)	0.68	13 (41.9)	127 (47.9)	0.53
Iron deficiency, n (%)[*]	38 (34.2)	397 (34.6)	0.94	11 (15.7)	149 (22.1)	0.22	34 (33.3)	222 (42.9)	0.07	11 (36.7)	104 (35.5)	0.90	4 (33.3)	61 (57.0)	0.12
Anemia, n (%)[†]	36 (33.6)	493 (41.2)	0.13	32 (46.4)	377 (56.3)	0.12	n/a	n/a	NA	24 (77.4)	263 (88.0)	0.10	19 (63.3)	172 (65.4)	0.82
IDA, n (%)[‡]	15 (14.7)	197 (17.9)	0.42	8 (11.8)	109 (16.3)	0.33	n/a	n/a	NA	8 (29.6)	88 (31.3)	0.86	4 (36.4)	41 (39.1)	0.57
Hemoglobin, g/dL	11.2 (1.1)	11.0 (1.1)	0.26	10.8 (1.1)	10.5 (1.1)	0.12	n/a	n/a	NA	9.8 (1.1)	9.5 (1.1)	0.10	10.6 (1.1)	10.2 (1.2)	0.34
MCHC, g/dL	33.0 (1.0)	32.9 (1.0)	0.72	n/a	n/a	NA	n/a	n/a	NA	n/a	n/a	NA	33.5 (1.1)	32.4 (1.1)	0.001
MCV, fL	70.6 (1.1)	71.1 (1.1)	0.51	75.3 (1.1)	75.6 (1.1)	0.72	n/a	n/a	NA	n/a	n/a	NA	76.5 (1.1)	73.5 (1.1)	0.07
Haptoglobin, µmol/L	n/a	n/a	NA	71.1 (3.5)	69.6 (3.0)	0.04	n/a	n/a	NA	n/a	n/a	NA	n/a	n/a	NA
Ferritin, µg/L	20.9 (2.4)	19.6 (2.9)	0.54	29.4 (2.5)	24.8 (2.4)	0.13	17.4 (2.5)	14.5 (2.6)	0.08	22.1 (3.0)	22.0 (2.3)	0.98	19.2 (3.8)	12.2 (3.4)	0.23
Hepcidin, µg/L	7.2 (3.2)	6.7 (3.3)	0.60	12.2 (4.0)	11.2 (4.7)	0.68	9.4 (2.9)	7.4 (3.6)	0.08	6.0 (4.3)	5.3 (4.2)	0.62	3.7 (4.6)	3.8 (3.9)	0.93
sTfR, mg/L	6.6 (1.9)	6.8 (2.0)	0.80	3.4 (1.5)	3.4 (1.4)	0.99	10.4 (1.5)	10.8 (1.5)	0.36	16.3 (1.8)	17.9 (1.7)	0.36	16.7 (1.7)	14.9 (1.5)	0.37
Iron, µmol/L	n/a	n/a	NA	9.0 (1.7)	8.6 (1.6)	0.46	n/a	n/a	NA	6.4 (1.6)	6.0 (1.8)	0.55	3.5 (2.3)	2.7 (3.1)	0.41
Transferrin, g/L	2.7 (1.3)	2.7 (1.3)	0.90	n/a	n/a	NA	2.5 (1.3)	2.7 (1.3)	0.06	2.7 (1.2)	2.7 (1.3)	0.81	2.9 (1.2)	3.0 (1.2)	0.37
TSAT, %	n/a	n/a	NA	13.3 (1.8)	12.8 (1.7)	0.58	n/a	n/a	NA	9.5 (1.8)	8.9 (2.0)	0.59	3.7 (2.6)	3.4 (3.4)	0.81
ZPP, µmol/mol heme	n/a	n/a	NA	97.4 (1.8)	114.6 (1.8)	0.03	n/a	n/a	NA	n/a	n/a	NA	n/a	n/a	NA
CRP/ACT, mg/L/g/L	1.1 (4.6)	1.4 (5.2)	0.11	0.5 (1.3)	0.4 (1.3)	0.12	0.8 (5.4)	0.9 (4.9)	0.86	2.2 (5.3)	2.9 (6.2)	0.41	1.2 (4.5)	1.1 (4.8)	0.86

Values are geometric means (±SD) for the various biomarkers. Proportions were compared between groups by Chi-square or Fisher’s exact test where appropriate, and continuous variables by Student´s t-test or Mann Whitney U test (Age). Q248H includes both heterozygotes and homozygotes.

[*]Iron deficiency defined as plasma ferritin < 12µg/L or < 30µg/L in the presence of inflammation (C-reactive protein >5mg/L α1-antichymotrypsin >0.6g/L in The Gambia) in children < 5 years or < 15µg/L in children >= 5 years

[†]Anemia defined as hemoglobin < 11g/dL

[‡]IDA, iron deficiency anemia defined as iron deficiency and anemia

ACT, α1-antichymotrypsin; CRP, C-reactive protein; IQR, interquartile range; MCHC, mean corpuscular hemoglobin concentration; MCV, mean corpuscular volume; sTfR, soluble transferrin receptor; TSAT, transferrin saturation; ZPP, zinc protoporphyrin; n/a, not available; NA, not applicable

Table S3. Estimates of the effect of Q248H heterozygotes and homozygotes on iron status and anemia.

	Q248H het		Q248H hom		Q248H WT		WT vs Het		WT vs Hom		Dominant model adjusted for HbAS	
**Category**	**total**	**n (%)**	**total**	**n (%)**	**total**	**n (%)**	**OR (95% CI)**	**P value**	**OR (95% CI)**	**P value**	**OR (95% CI)**	**P value**
Anemia*	229	107 (46.7)	8	4 (50.0)	2429	1305 (53.7)	0.75 (0.57, 0.98)	0.037	0.85 (0.21, 3.43)	0.82	0.69 (0.51, 0.92)	**0.012**
Iron deficiency†	313	97 (31.0)	12	1 (8.3)	2740	933 (34.1)	0.80 (0.62, 1.04)	0.094	0.16 (0.02, 1.28)	0.08	0.77 (0.59, 0.99)	**0.042**
Iron deficiency anemia‡	201	35 (17.4)	7	0 (0.0)	2155	435 (20.2)	0.80 (0.54, 1.18)	0.25	N/A	N/A	0.76 (0.51, 1.12)	0.16
Inflammation§	63	321 (19.6)	3	13 (23.1)	1815	587 (20.9)	0.93 (0.69, 1.24)	0.62	1.19 (0.33, 4.37)	0.79	0.93 (0.70, 1.25)	0.65
**Biomarkers**	**total**	**Geometric mean (SD)**	**total**	**Geometric mean (SD)**	**total**	**Geometric mean (SD)**	**Estimate (95% CI)**	**P value**	**Estimate (95% CI)**	**P value**	**Estimate (95% CI)**	**P value**
Hemoglobin, g/dL	229	10.81 (1.15)	8	10.72 (1.19)	2429	10.59 (1.16)	0.21 (0.01, 0.41)	0.037	0.17 (-0.85, 1.18)	0.75	0.23 (0.04, 0.42)	**0.019**
MCHC, g/dL	128	33.08 (1.04)	3	34.22 (1.03)	1406	32.87 (1.04)	0.25 (0.03, 0.48)	0.028	1.10 (-0.30, 2.51)	0.12	0.15 (-0.07, 0.38)	0.19
MCV, fL	200	72.91 (1.12)	5	75.77 (1.07)	2119	72.76 (1.12)	0.12 (-0.92, 1.16)	0.82	1.73 (-4.56, 8.02)	0.59	-0.03 (-1.08, 1.02)	0.96
Haptoglobin, µmol/L	49	131.95 (1.99)	1	212.78 (N/A)	537	109.32 (1.87)	0.19 (0.01, 0.37)	0.039	0.57 (-0.63, 1.77)	0.35	0.19 (0.01, 0.37)	0.041
Ferritin, µg/L	313	20.53 (2.54)	12	29.41 (2.18)	2740	20.03 (2.84)	0.07 (-0.05, 0.19)	0.25	0.42 (-0.15, 1.00)	0.15	0.09 (-0.03, 0.20)	0.14
Hepcidin, µg/L	306	8.15 (3.51)	13	13.25 (2.76)	2761	7.33 (3.87)	0.12 (-0.04, 0.29)	0.14	0.54 (-0.20, 1.28)	0.15	0.13 (-0.03, 0.30)	0.10
sTfR, mg/L	312	7.65 (2.03)	13	8.90 (1.87)	2766	7.38 (2.15)	-0.04 (-0.13, 0.05)	0.33	0.09 (-0.33, 0.50)	0.68	-0.07 (-0.16, 0.01)	0.087
Iron, µmol/L	42	5.27 (1.90)	3	8.70 (1.98)	414	4.83 (2.35)	0.12 (-0.12, 0.37)	0.31	0.32 (-0.56, 1.21)	0.47	0.14 (-0.10, 0.37)	0.25
Transferrin, g/L	247	2.65 (1.27)	11	2.04 (1.46)	2122	2.70 (1.27)	-0.04 (-0.12, 0.04)	0.37	-0.56 (-0.91, -0.21)	0.002	-0.06 (-0.13, 0.02)	0.16
TSAT, %	37	7.34 (2.08)	3	19.15 (1.66)	390	6.95 (2.62)	0.04 (-0.24, 0.32)	0.77	0.70 (-0.27, 1.67)	0.16	0.09 (-0.18, 0.36)	0.52
ZPP, µmol/mol heme	64	97.99 (1.81)	2	81.22 (1.29)	618	114.63 (1.77)	-0.16 (-0.30, -0.02)	0.023	-0.30 (-1.05, 0.46)	0.44	-0.19 (-0.33, -0.04)	**0.011**

* Anemia was defined as hemoglobin < 11g/dL.
† Iron deficiency was defined as plasma ferritin < 12µg/L or < 30µg/L in the presence of inflammation in children < 5 years or < 15µg/L in children >= 5 years.
‡ Iron deficiency anemia was defined as iron deficiency with anemia.
§ Inflammation was defined as C-reactive protein >5mg/L or α1-antichymotrypsin >0.6g/L in The Gambia.
ACT, α1-antichymotrypsin; CRP, C-reactive protein; MCHC, mean corpuscular hemoglobin concentration; MCV, mean corpuscular volume; N/A, not applicable; sTfR, soluble transferrin receptor; HbAS, sickle cell trait; TSAT, transferrin saturation; ZPP, zinc protoporphyrin

Table S4. Estimates of the effect of Q248H heterozygotes and homozygotes on severe malaria and bacteremia status. Estimates are presented adjusted for principal components, and additionally adjusted for genotype at the sickle-cell allele HbS (encoded by rs334)

**Outcome and model**	**Sample set**	**All**	**Q248H het**	**Q248H hom**	**WT**	**RR / OR* (95% CI)**	**P value**	**RR / OR* (95% CI)** adjusted for HbS genotype	**P value**
Severe malaria									
General (2-parameter model; heterozygote and homozygote versus WT)									
	Meta-analysis	4888/10179 (48.0%)	561/1247 (45.0%)	29/56 (51.8%)	4298/8876 (48.4%)	0.90 (0.80, 1.02)† 1.33 (0.77, 2.30)‡	0.13	0.91 (0.80, 1.03)† 1.37 (0.78, 2.42)‡	0.18
	(The Gambia)	2419/4910 (49.3%)	214/461 (46.4%)	11/18 (61.1%)	2194/4431 (49.5%)	0.86 (0.71, 1.05)§ 1.64 (0.63, 4.31)**	0.19	0.86 (0.70, 1.05)§ 1.55 (0.56, 4.24)**	0.22
	(Malawi)	1023/2345 (43.6%)	162/398 (40.7%)	12/21 (57.1%)	849/1926 (44.1%)	0.86 (0.69, 1.07)§ 1.58 (0.66, 3.77)**	0.23	0.88 (0.71, 1.11)§ 1.72 (0.70, 4.22)**	0.26
	(Kenya)	1446/2924 (49.5%)	185/388 (47.7%)	6/17 (35.3%)	1255/2519 (49.8%)	0.99 (0.79, 1.23)§ 0.81 (0.29, 2.28)**	0.92	1.01 (0.81, 1.27)§ 0.87 (0.30, 2.53)**	0.97
Recessive									
	Ghana	601/1803 (33.3%)	65/208 (31.3%)	3/8 (37.5%)	533/1587 (33.6%)	1.29 (0.33, 4.97)††	0.72		-
Bacteremia (Kenya only)									
General (2-parameter model; heterozygote and homozygote versus WT)									
	Overall	1536/4213 (36.5%)	214/580 (36.9%)	9/27 (33.3%)	1313/3606 (36.4%)	1.01 (0.84, 1.21)§ 0.92 (0.39, 2.00)**	0.97	1.01 (0.84, 1.21)§ 0.92 (0.39, 1.99)**	0.97
	S. pneumoniae	426/4213 (10.1%)	53/580 (9.1%)	4/27 (14.8%)	369/3606 (10.2%)	0.90 (0.66, 1.22)§ 1.65 (0.55, 4.95)**		0.89 (0.65, 1.22)§ 1.60 (0.53, 4.79)**	
	nontyphoidal *Salmonella*	180/4213 (4.3%)	31/580 (5.3%)	0/27 (0%)	149/3606 (4.1%)	1.31 (0.87, 1.95)§ -		1.30 (0.87, 1.95)§ -	
	E. coli	151/4213 (3.6%)	26/580 (4.5%)	0/27 (0%)	125/3606 (3.5%)	1.30 (0.85, 2.00)§ -	0.12‡‡	1.30 (0.85, 2.00)§ -	0.12‡‡
	H. influenzae	128/4213 (3.0%)	10/580 (1.7%)	2/27 (7.4%)	116/3606 (3.2%)	0.59 (0.32, 1.11)§ 2.62 (0.60, 1.15)**		0.59 (0.31, 1.10)§ 2.54 (0.58, 1.12)**	
	S. aureus	175/4213 (4.2%)	20/580 (3.4%)	0/27 (0%)	155/3606 (4.3%)	0.87 (0.54, 1.40)§ -		0.87 (0.54, 1.40)§ -	

* adjusted for 5 PCs (malaria analysis), 2 PCs & sex (bacteremia analysis)
† estimate from meta-analysis of heterozygote effect in case-control sets
‡ estimate from meta-analysis of homozygote (recessive) effect in case-control and trio sets
§ estimate of heterozygote effect in case-control set. Heterozygote and homozygote effects are jointly modeled.
** estimate of homozygote effect in case-control set. Heterozygote and homozygote effects are jointly modeled.
††Estimate of recessive effect in trio sets.
‡‡ Likelihood ratio test of multinomial regression model.

Table S5. Deviation from Hardy-Weinberg equilibrium for the variant causing the *FPN* Q248H mutation.

**Population**	**Observed Heterozygosity**	**Expected Heterozygosity**	**HWE[*] (*P*)**
ESN	0.11	0.12	0.324
GWD	0.12	0.11	0.278
LWK	0.05	0.05	1
MSL	0.06	0.06	1
YRI	0.09	0.09	1
Uganda	0.08	0.08	1
Burkina Faso	0.09	0.10	0.079
South Africa	0.15	0.15	0.811
Kenya	0.14	0.14	0.499
Ghana	0.12	0.12	1
Malawi	0.19	0.20	0.748
MKK	0.03	0.03	1

[*]HWE: Hardy-Weinberg equilibrium with statistical evidence of deviation calculated using Fisher's exact test

Table S6. Rare alleles present in populations included in the 1000 Genomes Phase 3.

Population	Number rare alleles*	Number filtered alleles
Africa†	1,687,002	3,805,147
East Asia‡	431,908	273,590
South Asia§	571,664	30,038
Europe**	14,761	14,761

*Rare alleles include those with frequencies between 0.01 and 0.09 in the populations alone

†For Africa populations filtered alleles were those with frequencies between 0.01 and 0.09 in Africa and 0.001 and 0.01 in America and absent in Europe and Asia (equivalent to the FPN mutation).

‡For East Asia populations filtered alleles were those with frequencies between 0.01 and 0.09 in East Asia and 0.001 and 0.01 in South Asia and absent in Europe, Africa and America.

§For South Asia populations filtered alleles were those with frequencies between 0.01 and 0.09 in South Asia and 0.001 and 0.01 in East Asia and absent in Europe, Africa and America.

**For Europe populations filtered alleles were those with frequencies between 0.01 and 0.09 in Europe and absent in Europe, Asia, Africa and America

Appendix A

The list of individuals involved in the Kenyan Bacteraemia Dataset generation and curation are as follows:
The Kenyan Bacteraemia study group

Principal Investigators: Adrian V S Hill (Chair), Thomas N Williams, J Anthony G Scott, Stephen J Chapman

Key Personnel: Anna Rautanen, Tara C Mills, Kirk Rockett, Anne W Ndungu, Vivek Naranbhai, Alex W Macharia, Sophie Uyoga, Carolyne Ndila, Neema Mturi, Patricia Njuguna, Shebe Mohammed, James A Berkley, Isaiah Mwangi, Salim Mwarumba, Barnes S Kitsao, Brett S Lowe, Susan C Morpeth, Iqbal Khandwalla

The Kilifi DNA extraction Group: Alex W Macharia, Sophie Uyoga, Herbert Opi, Carolyne Ndila, Emily Nyatichi, Prophet Ingosi, Barnes Kitsao, Clement Lewa, Johnstone Makale, Adan Mohamed, Kenneth Magua, Mary Njoroge, Gideon Nyutu, Ruth Mwarabu, Metrine Tendwa and Thomas N Williams.

The Kilifi Bacteraemia Surveillance Group: Ismail Ahmed, Samuel Akech, Alexander Balo Makazi, Mohammed Bakari Hajj, Andrew Brent, Charles Chesaro, Hiza Dayo, Richard Idro, Patrick Kosgei, Kathryn Maitland, Kevin Marsh, Laura Mwalekwa, Shalton Mwaringa, Charles Newton, Mwanajuma Ngama, Allan Pamba, Norbert Peshu, Anna Seale, Alison Talbert and Thomas N Williams.

Wellcome Trust Case Control Consortium 2

Management Committee: Peter Donnelly (Chair), Ines Barroso (Deputy Chair), Jenefer M Blackwell, Elvira Bramon, Matthew A Brown, Juan P Casas, Aiden Corvin, Panos Deloukas, Audrey Duncanson, Janusz Jankowski, Hugh S Markus, Christopher G Mathew, Colin NA Palmer, Robert Plomin, Anna Rautanen, Stephen J Sawcer, Richard C Trembath, Ananth C Viswanathan, Nicholas W Wood

Data and Analysis Group: Chris C A Spencer, Gavin Band, Céline Bellenguez, Colin Freeman, Garrett Hellenthal, Eleni Giannoulatou, Matti Pirinen, Richard Pearson, Amy Strange, Zhan Su, Damjan Vukcevic, Peter Donnelly

DNA, Genotyping, Data QC and Informatics Group: Cordelia Langford, Sarah E Hunt, Sarah Edkins, Rhian Gwilliam, Hannah Blackburn, Suzannah J Bumpstead, Serge Dronov, Matthew Gillman, Emma Gray, Naomi Hammond, Alagurevathi Jayakumar, Owen T McCann, Jennifer Liddle, Simon C Potter, Radhi Ravindrarajah, Michelle Ricketts, Matthew Waller, Paul Weston, Sara Widaa, Pamela Whittaker, Ines Barroso, Panos Deloukas.

Publications Committee: Christopher G Mathew (Chair), Jenefer M Blackwell, Matthew A Brown, Aiden Corvin, Chris C A Spencer
